# Unbiased Screening Identifies Functional Differences in NK Cells After Early Life Psychosocial Stress

**DOI:** 10.3389/fimmu.2021.674532

**Published:** 2021-07-30

**Authors:** Sara B. Fernandes, Neha D. Patil, Sophie Meriaux, Maud Theresine, Claude. P. Muller, Fleur A. D. Leenen, Martha M. C. Elwenspoek, Jacques Zimmer, Jonathan D. Turner

**Affiliations:** ^1^Department of Infection and Immunity, Luxembourg Institute of Health, Esch-sur-Alzette, Luxembourg; ^2^Doctoral School in Systems and Molecular Biomedicine, University of Luxembourg, Esch-sur-Alzette, Luxembourg

**Keywords:** early life stress, NK cell, maternal deprivation, immune system, natural killer cells

## Abstract

Early Life Adversity (ELA) is closely associated with the risk for developing diseases later in life, such as autoimmune diseases, type-2 diabetes and cardiovascular diseases. In humans, early parental separation, physical and sexual abuse or low social-economic status during childhood are known to have great impact on brain development, in the hormonal system and immune responses. Maternal deprivation (MD) is the closest animal model available to the human situation. This paradigm induces long lasting behavioral effects, causes changes in the HPA axis and affects the immune system. However, the mechanisms underlying changes in the immune response after ELA are still not fully understood. In this study we investigated how ELA changes the immune system, through an unbiased analysis, viSNE, and addressed specially the NK immune cell population and its functionality. We have demonstrated that maternal separation, in both humans and rats, significantly affects the sensitivity of the immune system in adulthood. Particularly, NK cells’ profile and response to target cell lines are significantly changed after ELA. These immune cells in rats are not only less cytotoxic towards YAC-1 cells, but also show a clear increase in the expression of maturation markers after 3h of maternal separation. Similarly, individuals who suffered from ELA display significant changes in the cytotoxic profile of NK cells together with decreased degranulation capacity. These results suggest that one of the key mechanisms by which the immune system becomes impaired after ELA might be due to a shift on the senescent state of the cells, specifically NK cells. Elucidation of such a mechanism highlights the importance of ELA prevention and how NK targeted immunotherapy might help attenuating ELA consequences.

## Introduction

Early life adversity (ELA), which means stressful events occurring in the first 1000 days of life (1–3), plays a major role in adult-onset illness (4–6). These stressful events include a series of negative situations such as poor socio-economic status, parental mental disease, abandonment and/or institutionalization. Exposure to ELA has long-lasting effects on both mental and physical health as well as having negative behavioral consequences. It is associated with an increased risk of developing cardiovascular diseases (7), asthma (8), cancer (9) and mental disorders such as depression and anxiety later in life in both humans and in rodent models (10–14). There is now growing clinical and pre-clinical evidence that ELA and the associated negative health risk behaviors act through an altered immune system to induce the later-life disease risk (15–19).

Development of the immune system starts in early gestation. Innate immune cells such as monocytes, neutrophils and NK cells appear in the first trimester of pregnancy. Adaptive immune cells (T and B-lymphocytes), emerge around the start of the second trimester (20–22). During the pre- and perinatal period, this development is highly affected by several maternal factors such as obesity (23), malnutrition (24, 25), anxiety (19, 26–28) and smoking (29, 30), but also environmental factors such as parturition (31, 32), breastfeeding (33, 34) and antibiotic treatment (35). Alterations in specific cellular subsets in the immune system resulting from ELA have been widely documented in clinical studies. Individuals subjected to parental separation and subsequent adoption displayed a higher activation state of the immune system, with decreased levels of circulating central memory T cells and CD8+ T regulatory cells (18). Teenagers who suffered from childhood maltreatment, such as sexual and physical abuse, physical and emotional neglect showed increased circulating levels of NK and NKT cells after ELA (36). In addition, blood levels of the inflammatory marker C - reactive protein (CRP) in teenagers who had a low social-economic status as children was found to be increased when compared to individuals not exposed to early stress (37). Individuals with poor maternal care and harsh discipline during childhood also presented increased levels of CRP in the blood (38). Studies with rhesus monkeys, early isolated from their mothers at early age, also show a significant decrease in the CD4+/CD8+ ratio and increase in the circulating levels of NK cells (39). Even though some animals studies do not show any difference in the cell number after maternal separation (40), others document a decrease of the CD8+ T cells with subsequent increase of the CD4+/CD8+ ratio (13), opposite to what was documented in monkeys. Furthermore, prenatal exposure to alcohol lead to an increase in the CRP serum levels, indicating an inflammatory state of the immune system (41), similarly to what was previously observed in clinical studies (37, 38). Despite these numerous investigations, the literature is still lacking an unbiased overview of the complete cellular immune system.

Although the mechanisms through which these events occur are still not fully understood, increasing evidence shows that the mechanism by which ELA influences the function of CD8+ T cells and, consequently, viral responses, may be through the HPA axis (42). The neuro-endocrine axis plays an important role in the adaptive response to stress. When facing insults, corticotropin-releasing factor (CRF) is released from the hypothalamus, which in turn stimulates the production and release of adrenocorticotropin (ACTH). This hormone’s main target is the adrenal cortex where the production and release of glucocorticoids (GCs) happens. Release of GCs into the bloodstream will trigger the adaptive mechanisms, in a negative feedback manner (43, 44). Stress events in an early period of life are known to have an impact in the HPA axis, programming its effects and responses in adulthood. This leads to decreasing levels of blood corticosterone and cortisol, which consequently affects the response of the peripheral immune system, leading to compromised viral responses (13, 45, 46). Such dysregulation of the HPA axis is thought to occur through the glucocorticoid receptor (GR), by regulation of gene transcription and negative feedback on the HPA axis, which in turn decreases the expression of certain cytokines (47). Clinical studies show an association between increased GR1F promoter methylation and ELA (48, 49). However GR/GC signaling remains undisturbed after ELA despite a slight increase in GR1F promoter methylation (50), raising doubts as to the importance of single-digit changes in promoter methylation levels (51).

ELA also accelerates immunosenescence, the natural aging process by which the immune cells begin to deteriorate and lead to weakened immune responses (52). Immunosenescence is accelerated not only after exposure to ELA (53), but also with depression after physical injury (54). T cells are strongly affected. Naïve T cell numbers decrease while memory T cell and terminally differentiated effector T cell (TEMRA) numbers increase, with concurrent telomere shortening (55–58). Furthermore, these cells have decreased expression of the co-stimulatory CD28 molecule and increased expression of the glycopeptide CD57, which leads to increased cytotoxicity and decreased proliferative capacity (53, 55, 59, 60). This increase in T cell senescence after ELA has been reported to be influenced by the exposure to and subsequent reactivation of cytomegalovirus (CMV), as levels of CD57+ cells are increased in patients seropositive for CMV (53, 61–63). Moreover, CMV in ELA individuals was recently reported to be linked to the presence of certain gut bacteria and CD8+CD57+ cells (64), suggesting an impact of ELA through the immune-brain-gut axis.

Although it is not as well documented as for T cells, immunosenescence also occurs in other cell types, such as B (65, 66) and NK cells (67, 68). The expression of CD57 in natural killer cells does not necessarily mean they are senescent but rather that they reached a higher maturation state, which is accompanied by functional changes similar to those observed in senescent T cells: less proliferation and higher cytotoxic capacity (28, 67, 69). Moreover, NK cells are clearly involved in the response to CMV infections (70, 71) and links between NK cells, CMV and immunosenescence are starting to emerge (69, 70, 72).

Using an unbiased screening tool for flow cytometry data visualization, viSNE (73), this study provides a detailed description of the overall immune changes induced in the rat maternal deprivation (MD) model of ELA, identifying unexpected, but clear changes in NK cell properties. Furthermore, we describe the functional profile of NK cells, showing a shift in the maturity and cytotoxic capacities. We validated the NK cell phenotype in samples from our EpiPath ELA cohort (53, 74). This cohort consists of young adults (average age 24) institutionalized or otherwise separated from their biological parents at birth and adopted in early childhood (mean age of adoption 4.5 months) together with control participants in their natal families, all brought up in Luxembourg under similar societal and socioeconomic conditions.

## Material and Methods

### Human Samples

Peripheral blood mononuclear cells (PBMCs) from individuals that had experienced ELA in the form of institutionalization and subsequent adoption were obtained from our previously published EpiPath cohort (18, 50, 74). Briefly, participants aged between 18 and 35 years old with a prior history of ELA (institutionalization followed by adoption) or raised by their natural parents were recruited in Luxembourg between 2014 and 2016. Baseline EDTA anti-coagulated blood samples were drawn at a fixed time (11 am). Peripheral blood mononuclear cells were isolated by Ficoll-Paque density gradient centrifugation as previously reported (53). and stored in liquid nitrogen until analyzed. All participants provided written informed consent, and the study was performed in accordance with the Declaration of Helsinki. The study was approved by the Luxembourg National Research Ethics Committee (CNER, No 201303/10 v1.4) and the Ethics Review Panel (ERP, University of Luxembourg, No 13-002).

### Animals

Ten to twelve week old 2-day timed-pregnant Wistar rats were obtained from Janvier Labs (Le Genest-Saint-Isle, France). Pregnant dams were housed in groups of 3 in 48 × 37.5 × 21 cm clear plastic isolator cages (Tecniplast, Varese, Italy) under a conventional 12-h light-dark cycle at 21°C and 49-54% relative humidity with food and water provided ad libitum. During pregnancy only routine husbandry was performed. Nesting material was provided for all females from gestational day (GD) 16 onwards and the cage was not changed between GD17 and post-natal day (PND) 2. Litters were naturally delivered between days 21-23 of gestation and size was adjusted to 12 pups/dam. Dams were randomly assigned to give birth to pups for one of the following groups (one condition per litter; two litters per group): 3 hours Maternal Deprivation from PND2 to PND14 (MD_180_), 15 minutes Maternal Deprivation from PND2 to PND14 (MD_15_) and no separation (CTR). Study outcomes are thus from two independent experiments. The experiments were carried out in accordance with the European Union directive 2010/63/EU as incorporated in Luxembourgish law for the care and use of laboratory animals. The study protocol was approved by the local Animal Welfare Structure (DII-2017-18).

### Rat Maternal Deprivation

Pups from both MD groups underwent a separation from the dam at a fixed time every day (MD_180_: 9 am - 12 am, MD_15_: 9 am - 9:15 am) from PND 2 to PND 14. Separated pups were placed in a clean bedding-free cage and maintained at 33°C in a heated vented animal cabinet (Noroit, France). At the end of the daily separation period, pups were returned to their mothers in the original home cage. Control litters were only handled for regular husbandry (e.g. cage cleaning) and otherwise left undisturbed until weaning. All animals were weaned on PND21, and subsequently housed (2 to 3 per cage) by sex and experimental group, and only received regular husbandry until further experiments.

### Rat Restraint Stress

All animals underwent a 1-hour restraint stress on PND49 +/- 1 day. Restraint stress was performed between 9 and 12 am during the inactive (light) phase. Animals were immobilized in a 50mm diameter dark grey PVC tube, closed at the front and with an adjustable lock in the back. Breathing of the animals was controlled during the whole procedure.

### Rat Corticosterone and Glucose Levels

Blood samples were drawn from the tail vein using a SAFETY Blood Collection/Infusion Set (Greiner Bio-One, Germany), immediately on being placed in the restrainer and in the minutes preceding their release. At the same time, a single blood drop was used to measure glucose levels, using an electronic glucometer (Accu-Chek, Roche). All blood samples were centrifuged at 2000 x g for 5 minutes and the plasma collected and stored at -80°C, until further analysis. Plasma corticosterone levels were measured by ELISA (IBL International, Hamburg, Germany), according to the manufacturer’s instructions. A 4-parameter curve was fitted to the calibrator sample OD values; sample concentrations were calculated and, for glucose, presented as delta values (values after stress – values before stress).

### Rat Immunophenotyping

At PND56 animals were euthanized by CO2 inhalation and cardiac puncture was performed post- mortem to collect blood. Post-mortem blood (100µL per animal) was used for immunophenotyping by flow cytometry (LSR Fortessa, BD Biosciences, NJ, USA). Cell surface specific antibodies (see **Supplementary Table 1**) were diluted in flow cytometry staining (FACS) buffer (1X PBS, 1% BSA, 2mM EDTA), added to each individual sample and incubated for 30 minutes, at 4°C in the dark. Subsequently, samples were washed three times (100µl, 4°C, 300 x g, 10 minutes, FACS buffer) and erythrocytes lysed with Lysis buffer (BD Biosciences) for 10 minutes at room temperature in the dark. Cells were fixed with fixation buffer (Invitrogen, CA, USA) for 1h, washed (100µl, 4°C, 300 x g, 10 minutes, FACS buffer) and permeabilized for 1 hour with permeabilization buffer (Invitrogen, CA, USA). Intracellular markers (**Supplementary Table 1**) were diluted in FACS buffer and added to the samples. After 30 minutes incubation (4°C, protected from light), the samples were washed three times (100µl, 4°C, 300 x g, 10 minutes) and re-suspended in FACS buffer for further analysis.

### Natural Killer Cell Phenotyping

NK cell phenotyping was performed on both rat splenocytes and human PBMCs. Single-cell splenocyte suspensions were prepared on the day of the sacrifice and stored in liquid nitrogen in FBS (Sigma Aldrich, MO, USA) containing 10% DMSO (Sigma Aldrich) until analyzed. On the day of the assay, vials were thawed at 37°C and washed with RPMI-1640 (Lonza, Basel, Switzerland) complemented with 10% FBS, 1% Penicillin/Streptomycin (Lonza), 1% Glutamine (Lonza) and 50µM of β-mercaptoethanol (Invitrogen). Cells were diluted to 106 cells/ml and 200µL aliquots distributed in 96 well plates, prior to incubation for 1 hour at 37°C, 95% humidity and 5% CO2. NK cell maturation state was assessed by flow cytometry (antibodies in **Supplementary Table 1**) as described above. Cell viability was measured using the Zombie NIR™ Fixable Viability Kit (Biolegend, San Diego, CA, USA).

### Natural Killer Cell Cytotoxicity Assays

The cytotoxic response of rat NK cells was determined against YAC-1, a murine lymphoma cell line. Target cells were thawed and cultured in suspension in flasks with complete RPMI medium (RPMI-1640, 10% FBS, 1% Pen/Strep, 1% Glutamine, 1 mM HEPES, 50µM β-mercaptoethanol). Only cells in the exponential growth phase were used in the assays. Single-cell splenocyte suspensions were cultured for 72 hours in complete RPMI-1640, with 200U/mL of recombinant rat IL-2 (Sigma Aldrich), at 37°C, 95% humidity and 5% CO2. Before the challenge, YAC-1 cells were stained with 1µM Cell Trace Violet (CTV) in 1XPBS for 20 minutes and washed twice with 1X PBS. Similarly, human PBMCs from the EpiPath cohort (18) were cultured in complete medium with 200U/mL of recombinant human IL-2 (R&D Systems Inc., MN, USA) and left undisturbed overnight. For human NK cells, the cytotoxic response was determined against K562, a human myeloid leukemia cell line. Cells were cultured in suspension in flasks with complete DMEM (DMEM, 10% FBS, 1% Pen/Strep, 1% Glutamine, 1 mM HEPES) and only taken for the assays at the exponential growth phase. Before the assay, K562 were pre-incubated with 1µM CTV for 20 minutes and washed twice with complete RPMI-1640 (RPMI-1640, 10% FBS, 1% Pen/Strep, 1% Glutamine). Effector NK cells (E) and YAC-1 or K562 target cells (T) were plated at E:T ratios ranging from 1:1 to 100:1 for rat splenocytes and 1:1 to 25:1 for PBMCs, for four hours. Fifteen minutes before acquisition, 15µM of TO-PRO3 (Invitrogen, Karlsruhe, Germany) was added, to discriminate viable cells from dead cells (TO-PRO3+).

### Natural Killer Cell Degranulation Assay

Human PBMCs were cultured overnight in complete medium with 200U/ml of IL-2 and stimulated with CTV labelled K562 target cells at ratios of (E:T): 1:1, 5:1, 10:1 and 25:1 as described above. At the same time, anti-CD107a antibody was added to each well. After 1h incubation, 0.1µL of GolgiStop (BD Biosciences) was added per well and the plate was incubated at 37°C, 5% CO2 for a further three hours. Cells were washed with FACS buffer (10 minutes, 300 x g) and stained for NK cell surface markers (**Supplementary Table 1**) followed by intracellular staining for IFN-γ as described above.

### Flow Cytometry

A minimum of 50,000 events were recorded for all the experiments. Immunophenotyping, NK cell maturity and degranulation assays were performed on BD LSR Fortessa (BD BioSciences using FACSDiva software (BD BioSciences, version 8.0). The NK cytotoxicity assays were analyzed on a NovoCyte Quanteon Flow Cytometer (Agilent).

### Data Analysis

Flow cytometry data was analyzed with FlowJo (Tree Star, Ashland, OR, USA), visNE software (Cytobank, Inc., CA, USA) and Tableau (Seattle, WA, USA). After processing the raw data, 36 flow cytometry.fsc files (12 per experimental group) from the 12-colour initial panel were uploaded onto Cytobank and used to generate viSNE plots according to the following parameters: Events = 50.000; Channels = all 12 antibodies; Compensation = uncompensated; Iterations = 5000; Perplexity = 30. For the illustration menu, the gating of all channels was set for minimum of -2000 and the argument at 200. For further and more detailed analysis, FlowSOM was used with the default settings and all channels and files were selected. Event sampling was set at 50.000; Number of metaclusters at 10; Iterations at 10; and Number of clusters at 49. Results of this analysis were plotted into t-SNEs maps and cell populations were separated according to the presence of each cell marker across the different cell populations.

### Statistical Analyses and Data Presentation

Statistical analyses were performed in GraphPad Prism version 8.0.0 for Windows (GraphPad Software, San Diego, CA, USA) and FlowSOM (Cytobank, Inc., CA, USA). Tests used to assess statistical differences were One-way ANOVA (Tukey’s multiple comparisons test) or Two-way ANOVA (Dunnett’s or Sidak’s multiple comparisons tests), depending on the number of animals and parameters in the assay. Figures were subsequently generated using GraphPad Prism and Adobe Illustrator CS6 (version 16.00).

All biosecurity and institutional safety procedures were adhered to.

## Results

### Maternal Deprived Animals Subjected to Stress in Adulthood Have an Increased Physiological Response to Acute Stress

**Figure 1:**
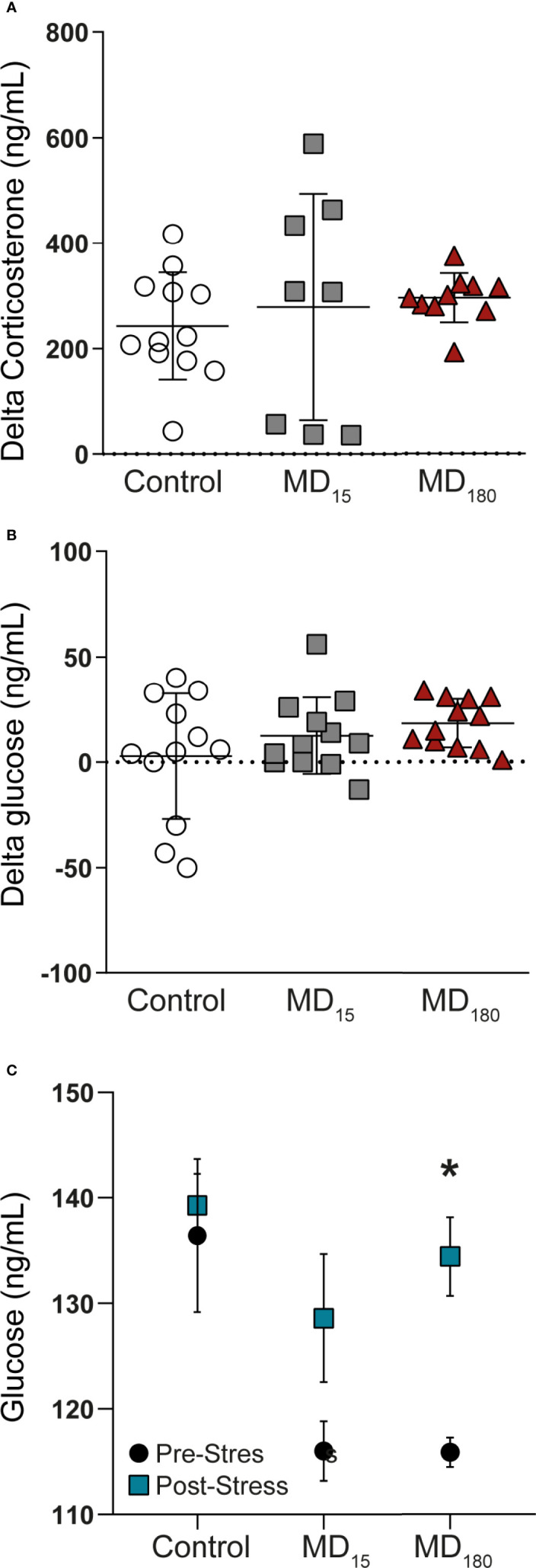
Acute stress in adulthood has no significant impact on the HPA axis of maternal deprived animals, but changes the glucose absolute levels. **(A)** Delta corticosterone levels; **(B)** Delta glucose levels; **(C)** Absolute glucose levels before and after an acute stress. Data is presented as mean ± SEM of 9 to 12 animals per group. Statistics: One-way and Two-way ANOVA. *p = 0.014.

At PND49, all animals were subjected to a restraint stress in order to evaluate HPA axis function. Corticosterone and glucose were measured from plasma and whole blood before and upon completion of the acute stressor, respectively. The restraint stress did not induce any significant changes in corticosterone and glucose levels in the two MD groups (**Figures 1A, B**; Tukey’s multiple comparisons test, p=0.81 for MD_15_ and p=0.6 for MD_180_ for corticosterone; p= 0.43 for MD_15_ and p=0.14 for MD_180_, for glucose). However, exposure to MD had a clear effect on the baseline glucose level, with exposure to either 15 or 180 minutes MD significantly lowering the baseline glucose level, compared to the control group [**Figure 1C**; MD_15_ 115.9 ± 1.4 *vs* 136.4 ± 7.2, p=0.0008; MD_180_ 116 ± 2.8 *vs* 136.4 ± 7.2, p=0.005, Dunnett’s multiple comparisons test)]. Although there was not a significant two-way interaction between the change in glucose and the groups, the stressor significantly increased the absolute glucose levels in the MD_180_ group (115.9mg/dL ± 1.4 *vs* 134.4mg/dL ±3.7, Sidak’s multiple comparisons test p=0.014) (**Figure 1C**). This shows an activation of gluconeogenesis in the liver (75) and release into the blood stream, indicative of the fight-or-flight response of a system in need of energy supply.

### Unbiased Immunophenotyping

**Figure 2:**
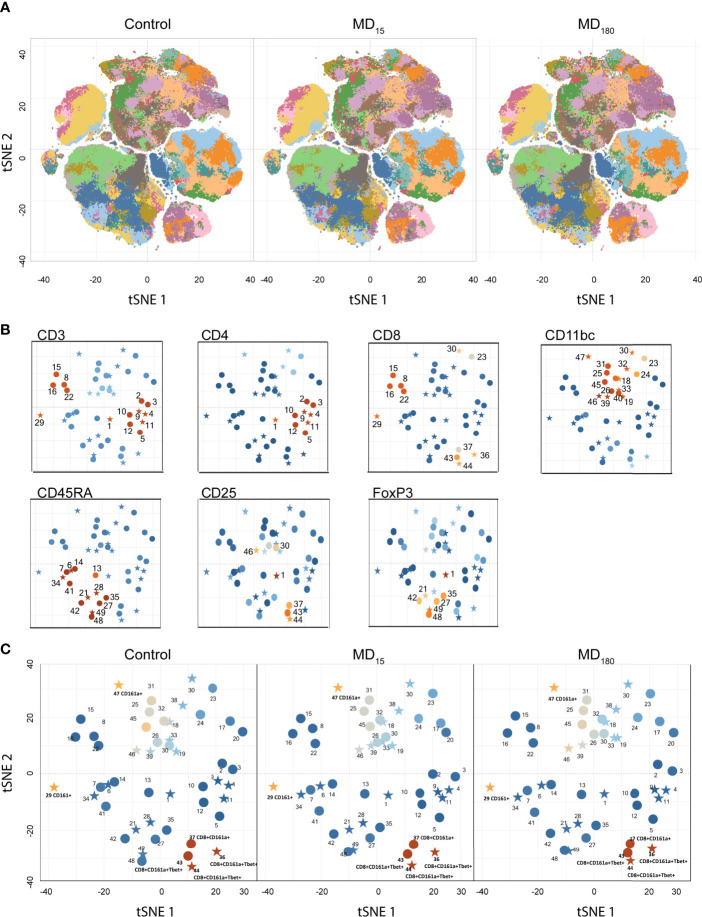
Unbiased immunophenotyping with viSNE. **(A)** visNE map obtained through cytobank with the markers a single 13-color antibody combination. Each panel represents the mean of all 12 animals in the control (left), MD_15_ (middle) and MD_30_ (right) groups; **(B)** Identification of different clusters by the principal rat cell surface markers – CD3 CD4, CD8, CD11b/c, CD45RA, FoxP3. Stars represent significantly different clusters, between the different treatment groups at an FDR corrected p < 0.05; **(C)** Clusters colored by CD161a (NK cell marker) intensity. Blue to orange: low to high expression of the marker.

Flow cytometry was performed for all animals at PND56. After basic data quality checks, t-SNE maps were generated from viSNE and flowSOM analysis. The t-SNE maps show clear differences in the clustering of the data through the abundance and spatial distribution of certain regions (**Figure 2A**). The map regions represent the different immune cell subsets from the animals, that were color defined based on the cell markers used (**Supplementary Figure 1**). In total, 49 different clusters were identified, twenty of which were found to be statistically different between both maternally separated groups and the control, according to the antibodies that define each cluster (**Supplementary Table 2**; **Figure 2B**). As seen in previous reports from other experimental paradigms (76–78), T (CD3^+^), T helper (CD4^+^) and T cytotoxic (CD8^+^) cells were the most clearly delineated populations within the viSNE plot and where we found the most significant changes after MD (**Supplementary Figure 1**). B cells (CD45RA^+^) (79, 80) together with clusters containing macrophages, dendritic (CD11b^+^) and T regulatory (CD25^+^, FoxP3^+^) cell types were also readily identified (**Supplementary Figure 2**). Surprisingly, some of the most significantly different clusters were associated with the CD161a cell marker, which is one of the primary cell surface markers for NK cells. (**Figure 2C**).

### Maternal Deprivation Induces Long-Term Changes in the Immune System

**Figure 3:**
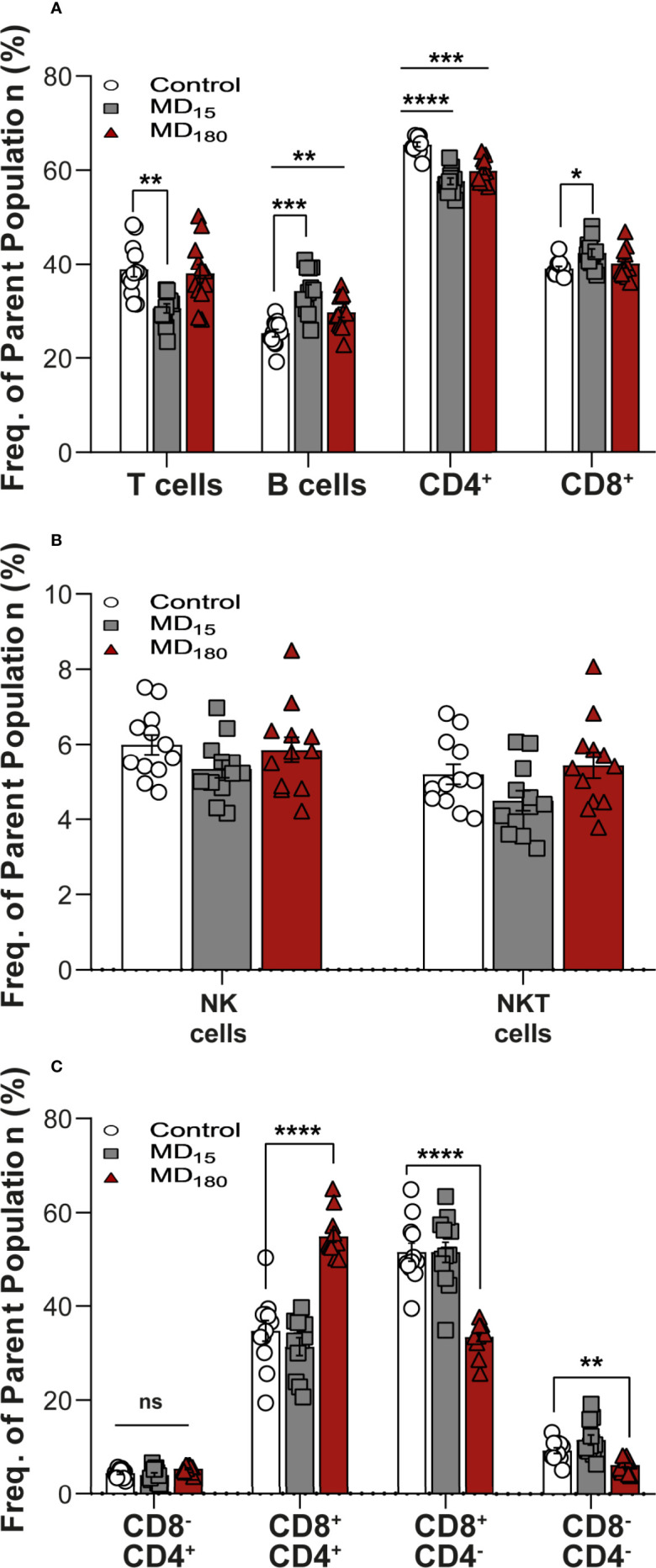
Maternal deprivation induces long-term changes in the immune system. **(A)** Adaptive immune cell population analysis. T cells gated as CD3+ from total lymphocytes. B cells gated as CD45RA+ gated from total lymphocytes; CD4+ and CD8+ gated from within the T cell population **(B)** expression levels of NK and NKT-like cells. NK and NKT cells were gated from total lymphocytes as the brighter population expressing CD161a+ and being CD3- or CD3+, respectively.; **(C)** Sub-gating of the NKT-cell population from panel **(B)** by CD4^+^ and CD8^+^ markers. Data is presented as mean ± SEM of 12 animals per group. Statistics: Two-way ANOVA; *p < 0.05; **p < 0.01; ***p < 0.001; ****p < 0.0001, NS, Not Significant. Representative Figures for flow data are included in the **Supplementary Material**.

The clusters identified in our viSNE analysis were examined in detail with FlowJo. The percentage of CD3^+^ T cells was significantly decreased in the animals subjected to 15 minutes of MD (30.5 ± 0.95, Dunnett’s multiple comparisons test, p=0.001), although no significant changes were found in the group separated for 3 hours, when compared to the control group (37.9 ± 1.98 *vs* 38.8 ± 1.62) (**Figure 3A**). B cells, on the other side, were found to be significantly increased in both groups (MD_15_: 34.2 ± 1.4, p<0.0008; MD_180_: 29.7 ± 1.04, p<0.0085), when compared to controls (25.3 ± 0.81) (**Figure 3A**). To further investigate how the immune system was impacted, we also looked at the different types of T cells. CD4^+^ helper T cells were found to be significantly decreased in both MD_15_ (57.7 ± 0.71, p<0.0001) and MD_180_ (59.8 ± 0.74, p=0.001), compared to the control group (65.5 ± 0.51). The cytotoxic CD8^+^ T cells were significantly increased in the MD_15_ group compared to controls (42.3 ± 0.86 *vs* 39.0 ± 0.48, p=0.022) but not in the MD_180_ (**Figure 3A**). Activated B cells and subsets of CD4^+^ and CD8^+^ T cells, which are involved in Th1, Th2 and Th17 types of responses, characterized by the transcription factors T-bet, GATA3 and RORγT, respectively, were also analyzed and quantified but did not produce significant changes upon early stress (data not shown), although assessed with anti-mouse antibodies.

### Levels of NK and NKT-Like Cells Changed After ELA

After our unbiased analysis with viSNE, we quantified the levels of both NK and NKT-like cells using a classical gating strategy (**Supplementary Figure 3**) in FlowJO. For the two populations, both MD groups did not show significant differences when compared to control (**Figure 3B**), but when separating the NKT cells into their functional subgroups defined by CD4 and CD8 expression (Seino and Taniguchi 2005), statistically significant differences appeared (**Figure 3C**). Double positive (CD4^+^CD8^+^) NKT-like cells were significantly increased in the MD_180_ (54.9 ± 1.31, Dunnett’s multiple comparisons test, p<0.0001), whereas 15 minutes of MD had no effect, when compared to the control group (34.77 ± 2.21). On the other hand, double negative (CD4^-^CD8^-^) NKT-like cells were significantly decreased in the MD_180_ group (6.19 ± 0.43, p<0.0036) but suffered no changes in the MD_15_ group, compared to controls (9.24 ± 0.62). Furthermore, CD8^+^ NKT-like cells were also found to be significantly decreased in the group separated for 3 hours in relation to the control group (33.4 ± 0.97 *vs* 51.5 ± 1.92, p<0.0001).

### Maternal Deprivation Changes the Maturation State of NK Cells

**Figure 4:**
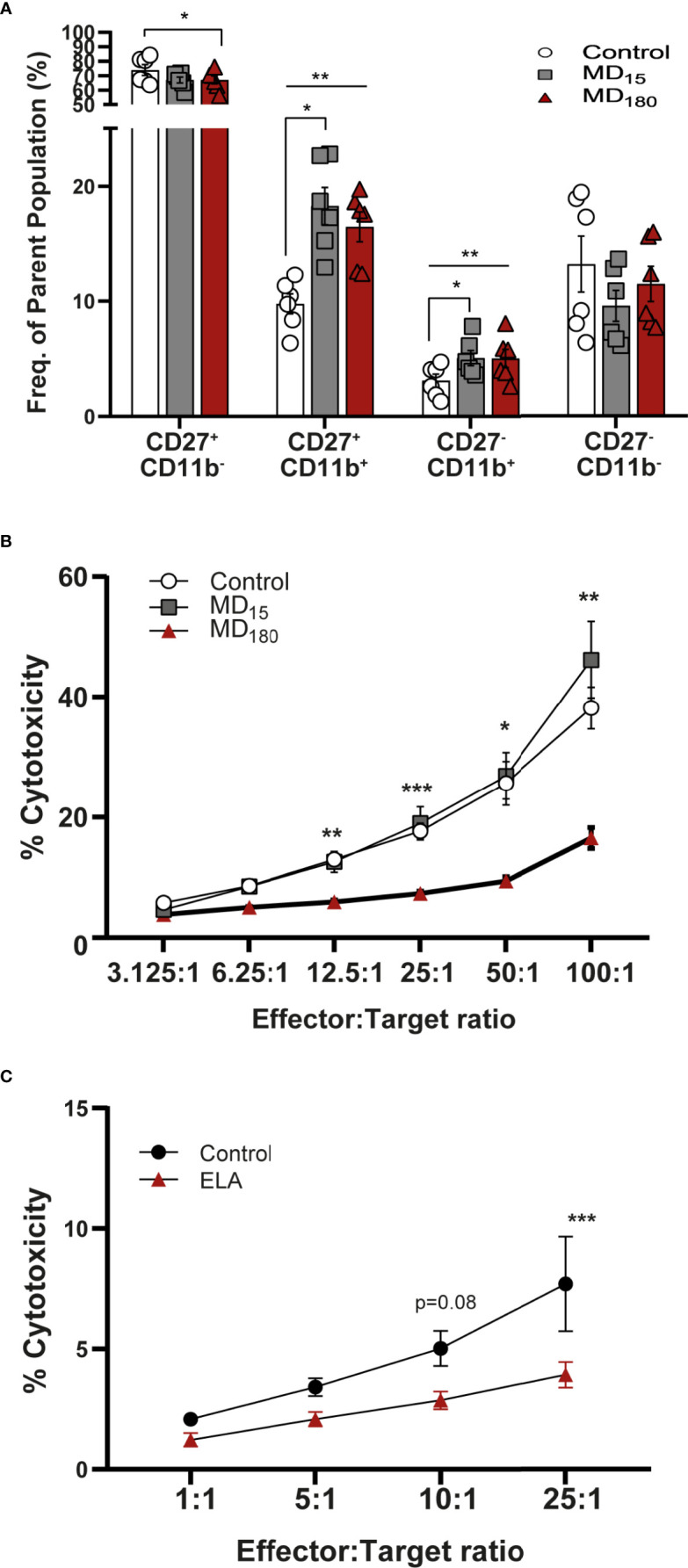
Early life separation induces functional changes in rat and human NK cells. **(A)** Maturation state of rat NK cells defined by CD27 and CD11b markers. Cells were gated from CD161a+/Nkp46+ NK cells; **(B)** Cytotoxic response of rat NK cells to YAC-1 cells; **(C)** Cytotoxic response of NK cells from institutionalized individuals to K562 cells. Data is presented as mean ± SEM of 6 animals per group or 14 donors per group. Statistics: Two-way ANOVA; *p < 0.05; **p < 0.01; ***p < 0.001. Representative Figures for flow data are included in the **Supplementary Material**.

To evaluate the effect of MD on NK cell functionality, we quantified the maturation state of these cells using the markers CD27 and CD11b (81–83). The process begins with no expression of either receptors (immature NK cells, iNK), followed by gain of CD27 and CD11b receptors, and ends with loss of CD27, representing the most mature NK cells (mNK) (gating strategy: **Supplementary Figure 4**). The double negative cell population (CD11b^-^, CD27^-^; Q4 from **Supplementary Figure 4**) does not appear to be influenced by our MD paradigm (**Figure 4A**). Following that, the NK cell population that gained CD27 but not CD11b (Q1 from **Supplementary Figure 4**) was shown to be significantly decreased in the MD_180_ group compared to the control group (67.0 ± 2.79 *vs* 73.9 ± 3.70, Dunnett’s multiple comparisons test, p=0.0119). The double positive (DP) population (Q2 from **Supplementary Figure 4A**) was significantly increased in both maternal deprived groups (MD_15_ 18.28 ± 1.61, p =0.019; MD_180_ 16.48 ± 1.29, p=0.0042) compared to the control group (9.76 ± 0.87). Finally, CD27^-^CD11b^+^ population (Q3 from **Supplementary Figure 4**), representing the most mature NK cells, was significantly increased in both maternal separated groups (MD_15_ 5.07 ± 0.66, p=0.0153; MD_180_ 5.03 ± 0.79, p=0.0023), compared to the control group (3.10 ± 0.56) (**Figure 4A**).

### Long Maternal Deprivation Changes the Cytotoxicity of NK Cells

The cytotoxic capacity of rat NK cells after MD was measured against the mouse target cell line YAC-1, as previously described in the literature (84). Cells from animals that underwent 3 hours of MD exhibited a significantly decreased cytotoxicity from E:T ratio 12.5:1 (5.94 ± 0.38, Dunnett’s multiple comparisons test, p=0.0032) to the highest E:T ratio, 100:1 (16.57 ± 1.77, p=0.0057), when compared to the cells of the group that did not suffer any type of early stress (12.99 ± 1.03; 38.16 ± 3.48) (**Figure 4B**). Animals that were maternal separated for 15 minutes displayed a similar response to the control group.

### NK Cell Changes Are Reproduced in the EpiPath ELA Cohort

The cytotoxic response of human NK cells from the EpiPath cohort was measured against K562 cells. Similarly to the rat, NK cells from the individuals that were exposed to ELA had a lower response than the cells from the control group, reaching statistical significance at the highest ratio (E:T, 25:1) (3.93 ± 1.78 *vs* 7.71 ± 6.52, Sidak’s multiple comparison test, p=0.0004) (**Figure 4C**). As previously seen in our study (53), increased titers of CMV could be associated with such a decrease in the cytotoxicity of NK cells. However, there is no statistical correlation in any of the ratios, between CMV titers and NK cytotoxicity (**Supplementary Figure 5**).

### Early Life Stress Reduces Degranulation of NK Cells in the EpiPath Cohort

**Figure 5:**
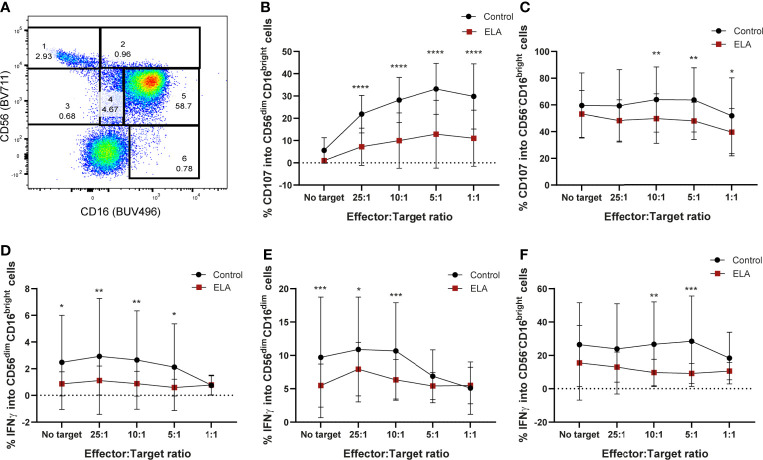
Maternal deprivation changes the degranulation capacity of the NK cells from ELA individuals. Cells were gated as lymphocytes from FSC-H, SSC-H; singlets from SSC-H, SSC-A; then from CD3-CD19- cells. **(A)** representative image of the NK cell population gating strategy: 1– CD56^bright^CD16^-^; 2 – CD56^bright^CD16^+^; 3- CD56^dim^CD16^+^; 4– CD56^dim^CD16^dim^; 5- CD56^dim^CD16^bright^; 6 – CD56^-^CD16^bright^; **(B, C)** expression of CD107a by populations 5 and 6; **(D–F)** expression of IFN-γ by populations 4, 5 and 6. Data is presented as mean ± SEM of 14 donors per group. Statistics: Two-way ANOVA; *p < 0.05; **p < 0.01; ***p < 0.001; ****p < 0.0001.

Similar to what was previously described (85, 86), six populations were obtained in the flow cytometry CD16 *vs* CD56 dot plot: CD56^bright^CD16^-^, CD56^bright^CD16d^im^, CD56dimCD16^bright^, CD56^-^CD16^bright^, CD56^dim^CD16^-^ and CD56^dim^CD16^dim^ (**Figure 5A**). In all these populations, the expression of CD107a and IFN-γ were measured. Although the majority of NK populations displayed less degranulation capacity in donors that suffered ELA (**Supplementary Figure 6**), only CD56^dim^, or CD56 negative NK cells (CD56^dim^CD16^dim^; CD56^-^CD16^bright^, CD56^dim^CD16^bright^), the most mature populations, reached statistical significance (**Figure 5**). CD56^dim^CD16^bright^ (population 5) NK cells from the control group displayed significantly higher levels of CD107a expression for all ratios, but did not show any differences when target cells were not presented (25:1 – 7.2 ± 8.4 *vs* 21.9 ± 8.4, p<0.0001; 10:1 – 9.9 ± 12.4 *vs* 28.2 ± 10.1, p<0.0001; 5:1 – 12.9 ± 15.2 *vs* 33.2 ± 11.4, p<0.0001; 1:1 – 11.04 ± 12.6 *vs* 29.8 ± 14.6, p<0.0001) (**Figure 5B**). The same was observed for the expression of IFN-γ, reaching statistical difference for all ratios except E:T 1:1 (No target – 0.87 ± 0.91 *vs* 2.5 ± 3.5, p=0.0171; 25:1 – 1.12 ± 1.08 *vs* 2.9 ± 4.3, p=0.0061; 10:1 – 0.88 ± 0.9 *vs* 2.7 ± 3.7, p=0.0072; 5:1 – 0.6 ± 0.63 *vs* 2.1 ± 3.2, p=0.0262) (**Figure 5D**). Double dim NK cells from ELA donors (population 4: CD56^dim^CD16^dim^) showed significantly decreased secretion of IFN-γ for all E:T ratios except 5:1 and 1:1, when compared to control donors [Sidak’s multiple comparisons test: No target - 5.48± 3.2 *vs* 9.71 ± 9.02, p=0.0009; 25:1 – 7.9 ± 4.01 *vs* 10.9 ± 7.9, p=0.033; 10:1 –6.3± 3.1*vs* 10.7 ± 7.2, p=0.0006] (**Figure 5E**). Expression of CD107a was not different between the groups (**Supplementary Figure 6**). Finally, the CD56^-^CD16^bright^ (population 6) NK cell population displayed significant differences at the E:T ratios 10:1 and 5:1 with lower expression in the ELA group for both CD107a (10:1 - 49.6 ± 18.6 *vs* 63.9 ± 24.4, p=0.0084; 5:1 – 47.9 ± 13.9 *vs* 63.6 ± 24.1, p=0.003)) and IFN-γ (10:1 -9.7 ± 7.9 *vs* 26.7 ± 25.4, p=0.0011; 5:1 – 9.2 ± 5.9 *vs* 28.5 ± 27.2, p= 0.0002) (**Figures 5C, F**). CD107a expression was also significantly decreased in the ELA group at the E:T ratio 1:1 (39.5 ± 17.7 *vs* 51.7 ± 28.5, p=0.0298) (**Figure 5C**).

## Discussion

In this study, we demonstrated how ELA, in the form of maternal separation, has a more widespread influence on the immune system than previously thought. To our knowledge, this is the first unbiased viSNE analysis linking ELA with clear changes in the overall immune profile and, more specifically, the first to provide specific mechanisms of maturation, senescence, and changes in cytotoxicity and degranulation profiles of NK cells after ELA. We initially examined the effect of ELA using the rat MD model. As previously reported by ourselves and others (87–89), the separation and deprivation of maternal care during this period has been associated with increased cognitive impairment, HPA-axis dysregulation and anxious-like behavior (13, 90–93).

Stress, in all types of forms, either in early life, adolescence or adulthood activates the HPA axis, leading to the release of cortisol that will bind to GRs and ultimately initiate a cascade of molecular and cellular events (94–96). GRs activation is reported to impact gene transcription (97) and to inhibit immune responses (47). However, we previously reported that this process is not always so clear-cut (50). We saw clear clinical consequences later in life, specifically in the immune system and in the development of chronic and psychological disorders (18, 53), however, these changes were not accompanied by alterations in the expression or response of GRs, although the HPA axis was hypo-responsive (50, 74). Although we did not directly assess the functioning of the GRs, our data confirm our previous report that HPA axis hormones and receptors might not be as intimately involved in the long-term consequences of early life stress as thought. Importantly, we were able to reproduce the immune phenotype previously seen in our EpiPath cohort (18), where CD8+ T cells were found to be more activated (18) and more senescent (53) than the cells from the individuals in the control group. In our paradigm, T cells (CD3+) and their subsets (CD4+ and CD8+) were significantly changed, with CD8+ T cells following the same trend as in our ELA (18), confirming the relevance of the MD model for the biological consequences of ELA. Furthermore, we expanded changes in the immune system to B and NK cells. Our unbiased viSNE analysis did not clearly distinguish NK and NKT-like cells, the latter being T cells that share and express NK cell receptors bridging the innate and adaptive immune responses that are implicated in tumor rejection, cardiovascular and neurological diseases (98–101). Little is known about the long-term effects on NK cells after early-life psychosocial stressors, although NK cell numbers have been reported to be impacted (102). However, our previous report (18), together with the data reported here, suggest that ELA has a minor impact on circulating NK cell numbers, but is accompanied by a higher activation state and a trend towards increased senescence. Our data suggest that the NK cells have a similar phenotype to the CD8 T cells, previously reported. We see a different secretion of CD107a and IFN-γ from the CD56dim NK cell subsets (CD56dimCD16dim and CD56dimCD16+), as well as from the CD56-CD16+. As discussed by Emily Mace (103) and others (104, 105), these subsets are thought to be the most differentiated ones, as loss of CD56 expression and acquisition of CD16 was proposed to be part of the maturation process. These results follow the increased expression of maturation markers observed in the rats. Altogether, this seems to indicate that, although immature cells in both adoptees and stressed animals are still functional, as they become more mature, they lose their functionality, both in terms of cytotoxicity and degranulation. In a similar way to the increased activation (CD25) and senescence (CD57) of CD8+ T cells, the NK cells appear to lose functionality as they mature, although unlike CD8 T cells, this was independent of CMV exposure and titers (18). It would appear that this mechanism is not applicable in NK cells, as there was no correlation between either CMV titers or seropositivity and NK cell activity. Furthermore, as there was no clear HPA axis phenotype, although there was a trend towards an increase in stress-induced gluconeogenesis, we conclude that the HPA axis is unaffected in our MD paradigm and, as such, cannot be responsible for the NK cell phenotype either. We saw clear differences in baseline glucose levels in both our MD groups. We initially hypothesized that this was a function of the altered HPA axis (106). However, it would appear that changes in the external environment did not induce changes in the HPA axis stress response nor in the overall glucocorticoid levels. As such, our recent hypothesis that the disturbed HPA axis fundamentally alters hepatic metabolism such as gluconeogenesis to mechanistically crystalize the early-life adversity associated risk of metabolic diseases may, in fact be erroneous, and that this may be independent of the HPA axis.

NK cells are known to be affected by current acute and chronic stress. The early work by Schedlowski et al. showed that acute stress in adulthood, in this case novice parachute jumpers, had significant changes in the circulating lymphocyte subsets as well as functional differences in NK cells immediately post-stress. We expanded on this to demonstrate the kinetics of NK cell redistribution throughout the day, coupled to the circadian HPA axis rhythm, though in the work of Schedlowski it appeared to be associated with noradrenaline levels (107, 108). Both studies suggested that this rapid mobilization of NK cells was a natural physiological reaction to an external stressor, in agreement with both their natural role as an immediate initiator of the immune response before adaptation starts, and as an evolutionary mechanism, preparing the body to fight injury or infection after encountering an acute stressor. Sympathetic nervous system control of NK cell action *via* noradrenaline has been suggested to be an advantage because of the speed with which the immune system can be primed to act after a stressful encounter, as well as the speed in which the priming can be terminated and homeostasis re-established (109) through the inflammatory reflex (110). There is a similar dearth of literature on the effects of chronic stress on NK cell functioning. In a similar manner to our observation of decreased NK cell functionality, chronic low-dose glucocorticoid administration reduced histone acetylation levels around promoters for two essential NK cell produced effectors: perforin and granzyme B. This was associated with lower mRNA transcript levels, lower protein levels, and NK cells were functionally impaired in a manner similar to what we report in both our rat model and in the EpiPath cohort. The lower perforin and granzyme B levels decreased their cytolytic activity. Inversely, the same administration regime increased histone acetylation of the IFN-γ and IL-6 promoters, up-regulating transcription and functional protein levels (111). The situation is, however, far from clear-cut. Children with current chronic stress from maternal mental health had higher levels of psychiatric symptoms as well as an increase in the number of illness episodes that was associated with increased NK cell cytotoxicity (102). However, none of the data available so far addresses the long-term effect of early life psychosocial stress and adversity, and the differences in NK cell functionality when the stressor is no longer present. In our previous report from the EpiPath cohort, multiple correction testing during our survey of the complete immune system meant that NK cells only narrowly missed significance (18), and the only other comparable study did not investigate NK cells (64, 112). Both of these studies reported that ELA induced a long-term immunosenescence and reduced T-cell functionality that was most probably due to continued re-activation of viruses such as CMV. It would seem logical that the exposure to a period of chronic stress in both models presented here has had a similar effect on NK cells. The two experimental systems show that once the period of ELA has resolved, NK cells are programmed with a long-term hypo-reactivity. As for acute stress preparing the NK cells to deal with an immediate infectious threat or potential wound, we suggest that this long-term hypo-reactivity is a similar evolution. The sensitive early life period would appear to have prepared the NK cells for an environment in which they can expect to be more regularly activated, and as such, to avoid any negative effects associated with NK cell secreted effector molecules or cytokines.

Although NK cells are often associated with a positive regulation of the immune response, they are also associated with the development of immunopathologies. In chronic hepatitis B virus infection, NK cells contribute to both liver inflammation and injury (113, 114), and aggravate and increase the lethality of bacterial infections in murine models (115, 116). NK cell activity was found to be impaired in patients that suffered from multiple sclerosis (117, 118), type-1 diabetes (119) and cardiovascular diseases (120–122), and found to sustain joint inflammation in rheumatoid arthritis patients (123); the risk of the latter three are all increased by exposure to ELA (16, 124–126). This raises the possibility of the long-term alteration of the NK cell phenotype underlying the pathophysiological effects of ELA. It may seem counterintuitive that we see an accumulation of NK cells of a more mature phenotype, but of lower functionality. This does, however, very clearly mirror our prior data on CD8+ T cells. Although it is not as well documented as for T cells, immunosenescence also occurs in other cell types, such as B (65, 66) and NK cells (67, 68). Our data suggests that, as suggested by Judge et al. (67), this corresponds to the accumulation of senescent NK-cells. Although NK cells have received far less attention, anergy, senescence and exhaustion are distinct dysfunctional entities that parallel the same processes in T cells and are all characterized by a significant reduction in both proliferation and NK-effector functions. There is the caveat that the expression of CD57 in natural killer cells does not necessarily mean they are senescent but rather that they reached a higher maturation state, which is accompanied by functional changes similar to those observed in senescent T cells: less proliferation and higher cytotoxic capacity (28, 67, 69). Furthermore, an alternative interpretation may be that at the single cell level ELA reduces NK cell effector functions, while at the population level the NK cells were more mature. This is somewhat confirmed by our previous report of ELA reducing activation CD69 levels on both total NK cells and CD56dimCD16+ NK cells. Unfortunately, in our previous report, this result was highly significant prior to, but not after, post-hoc multiple testing correction (18)

The limitations of our pre-clinical study include potential litter effects and the absence of a clear HPA axis phenotype. Similarly, the number of EpiPath participants analyzed was limited, however, based on the rat MD data, our power calculation suggested that to see the same phenotype in the cohort only 14 participants were required to have 80% power at alpha 0.05, which we largely exceeded. However, these are clearly outweighed by the reproduction of the functional NK cell phenotype in the EpiPath cohort. Furthermore, the identical phenotype in the MD model and the cohort allowed us to exclude the two most prominent mechanistic hypotheses from the literature – HPA axis control, and continual CMV reactivation. Nevertheless, ELA has a direct impact in the maturation state and later exhaustion of NK cells that may impair their activity and lead to uncontrolled reactions in adulthood.

It is clear that all cells of the immune system are not equally affected by ELA. Here, we have expanded our prior observation of T cell immunosenescence to a novel, unbiased examination of the immune system, identifying NK cells as functionally affected by ELA in both the rat MD model and in our human institutionalization – adoption cohort and NKT-like cells to be differently expressed in the rats. The immature NK cells appear to retain their functionality, however, as they mature towards CD56dim NK cell subsets and CD56- phenotype, their cytotoxic and degranulation potential are reduced. It is now evident that alterations in the HPA axis, either as stress-induced cortisol/corticosterone production or gluconeogenesis, are not responsible for the immune phenotype. The challenge is now to understand how ELA is inducing such changes and the role of both T cell and NK/NKT-like cells functional and expression loss in the long-term ELA-induced disease risk.

## Data Availability Statement

The original contributions presented in the study are included in the article/**Supplementary Material**. Further inquiries can be directed to the corresponding author.

## Ethics Statement

The studies involving human participants were reviewed and approved by Luxembourg National Research Ethics Committee (CNER, No 201303/10 v1.4) and the Ethics Review Panel (ERP, University of Luxembourg, No 13-002). The patients/participants provided their written informed consent to participate in this study. The animal study was reviewed and approved by local Animal Welfare Structure (DII-2017-18).

## Author Contributions

Conceptualization: SF and JT. Literature review: SF and JT. Data collection: SF, NP, SM, ME, FL, and MT. Data analysis: SF, NP, JZ, and JT. Manuscript writing and editing: SF, NP, JZ, and JT. All authors contributed to the article and approved the submitted version.

## Funding

This study was funded by the Fonds National de Recherche Luxembourg grants FNR-CORE (C16/BM/11342695 “MetCOEPs”), (C12/BM/3985792 “Epipath”) and the Ministry of Higher Education and Research of Luxembourg.

## Conflict of Interest

The authors declare that the research was conducted in the absence of any commercial or financial relationships that could be construed as a potential conflict of interest.

## Publisher’s Note

All claims expressed in this article are solely those of the authors and do not necessarily represent those of their affiliated organizations, or those of the publisher, the editors and the reviewers. Any product that may be evaluated in this article, or claim that may be made by its manufacturer, is not guaranteed or endorsed by the publisher.

## Abbreviations

MD, Maternal deprivation; NK cell, Natural killer cell; NKT, Natural killer-like T cell; PND, Post-natal day; ACTH, Adrenocorticotropin hormone; CRH, Corticotropin-releasing hormone; GCs, Glucocorticoids; HPA axis, Hypothalamic–pituitary–adrenal axis; CRP, C-reactive protein; PBMCs, Peripheral blood mononuclear cells; CMV, cytomegalovirus.
