# Supplementary material for: Unbiased Screening Identifies Functional Differences in NK Cells After Early Life Psychosocial Stress
Source: Front Immunol. 2021 Jul 30;12:674532. doi: 10.3389/fimmu.2021.674532 (PMC8363253; doi:10.3389/fimmu.2021.674532)
Supplement: Supplementary file 1 [file DataSheet_1.pdf]

Supplementary Table 1 – Description of the antibodies used for immunepehotyping.

IMMUNOPHENOTYPING			
Cell type	Antibody	Laser	Reference
T cells	CD3	FITC	BD 559975
T helper cell	CD4	BV510	BD 740138
T cytototxic cell	CD8	BUV395	BD 740257
B cells	CD45RA	PE Cy5	BD 561624
Activation marker	CD25	BV650	BD 742755
NK cells	CD161a	BV605	BD 744051
Macrophages, dendritic cells	CD11b/c	BB700	BD 746165
Activation marker B cells	RT1B	BV786	BD 744131
T regulatory cells	FoxP3	APC	BD 566527
Viability marker	Live/Dead	APC Cy7	Biolegend 423105
NK CELL MATURATION ASSAY			
**T cells**	CD3	PerCP Cy5.5	ThermoFisher 46-0030-82
**NK cell**	CD161a	BV605	BD BV605
**NK cell phenotypic marker**	NKp46 (CD335)	APC	Biolegend, 250808
**NK cells maturation marker**	CD27	BUV737	BD 612831
**NK cells maturation marker**	CD11b	PE	BD 562105
**Viability marker**	Live/Dead	APC-Cy7	Biolegend 423105
NK CELL CYTOTOXICITY ASSAY			
**Viability marker**	TO-PRO	APC	ThermoFisher Scientific T3605
**Viability marker**	Cell Tracer Violet	Pacific Blue	ThermoFisher Scientific C34571
**NK CELL DEGRANULATION ASSAY**			
**T cells**	CD3	BV510	BD 563109
**NK cell phenotypic marker**	CD19	PE-Cy5	Biolegend 302210
**NK cell phenotypic marker**	CD16	BUV496	BD 564653
**NK cell phenotypic marker**	CD56	BV711	BD 563169
**NK cell functional marker**	CD107a	FITC	Biolegend 328606
**Cytokine**	IFNγ	PE	BD 502509
**Viability marker**	Cell Tracer Violet	Pacific Blue	ThermoFisher Scientific C34571
Viability marker	Live/Dead	APC Cy7	Biolegend 423105

SupplementaryTable 2 - Clusters obtained from viSNE unbiased phenotyping. Association of the markers used with each cluster and p values obtained after statistical analysis.

		Cluster size				Post-hoc test		
**Cluster**	Markers	Control	MD15	MD30	P value	CTR vs MD_{15}	CTR vs MD_{180}	MD_{15} vs MD_{180}
**1**	$CD3^+$; $CD4^+$; $CD25^+$; $FoxP3^+$	1.95 +/-0.26	1.19 +/- 0.17	1.31 +/-0.18	1.34E-10	3.73E-10	1.86E-08	3.29E-01
**2**	$CD3^+$; $CD4^+$	2.71 +/-1.02	0.85 +/-0.47	2.11 +/-1.11	6.79E-05	5.39E-05	2.55E-01	5.10E-03
**3**	$CD3^+$; $CD4^+$	4.03 +/-1.01	2.56 +/-0.92	3.87 +/-1.25	3.23E-03	5.30E-03	9.31E-01	1.34E-02
**4**	$CD3^+$; $CD4^+$	3.78 +/-0.80	2.69 +/-0.72	2.62 +/-0.84	1.27E-03	4.91E-03	2.82E-03	9.77E-01
**5**	$CD3^+$; $CD4^+$	1.59 +/-0.77	1.31 +/-0.48	2.35 +/-1.08	1.06E-02	6.70E-01	7.41E-02	9.92E-03
**6**	$CD45RA^+$; $RT1B^+$	1.01 +/-0.45	1.87 +/-0.81	1.61 +/-0.46	3.95E-03	3.44E-03	4.86E-02	5.40E-01
**7**	$CD45RA^+$; $RT1B^+$	1.89 +/-0.79	2.21 +/-0.65	1.13 +/-0.28	4.39E-04	4.13E-01	1.25E-02	3.82E-04
**8**	$CD3^+$; $CD8^+$	3.46 +/-0.65	3.28 +/-0.56	2.47 +/-0.71	1.30E-03	7.56E-01	1.65E-03	1.10E-02
**9**	$CD3^+$; $CD4^+$	4.32 +/-0.37	2.22 +/-0.42	2.36 +/-0.37	2.48E-15	2.62E-14	1.73E-13	6.59E-01
**10**	$CD3^+$; $CD4^+$	2.28 +/-0.35	2.31 +/-0.76	3.21 +/-0.66	8.32E-04	9.93E-01	2.18E-03	3.00E-03
**11**	$CD3^+$; $CD4^+$	0.82 +/-0.22	2.31 +/-0.49	2.09 +/-0.82	3.26E-07	7.33E-07	1.27E-05	5.99E-01
**12**	$CD3^+$; $CD4^+$	ns	ns	ns	ns	ns	ns	ns
**13**	$CD45RA^+$	1.05 +/-0.40	2.11 +/-0.55	1.38 +/-0.62	8.57E-05	7.18E-05	3.01E-01	5.00E-03
**14**	$CD45RA^+$; $RT1B^+$	1.39 +/-0.62	2.96 +/-1.01	1.61 +/-0.47	1.50E-05	3.05E-05	7.55E-01	2.47E-04
**15**	$CD3^+$; $CD8^+$	ns	ns	ns	ns	ns	ns	ns
**16**	$CD3^+$; $CD8^+$	1.85 +/-0.28	2.57 +/-0.72	2.46 +/-0.87	2.71E-02	3.31E-02	7.98E-02	9.16E-01
**17**	$ROR\gamma T^+$	ns	ns	ns	ns	ns	ns	ns
**18**	$CD11b/c^+$	0.86 +/-0.20	1.87 +/-0.39	2.20 +/-0.55	4.47E-09	1.70E-06	5.57E-09	1.25E-01
**19**	$CD11b/c^+$	0.52 +/-0.30	1.96 +/-0.61	1.54 +/-0.55	1.92E-07	1.73E-07	6.70E-05	1.19E-01
**20**	$CD11b/c^+$	0.61 +/-0.16	0.51 +/-0.20	1.24 +/-0.98	8.27E-03	9.10E-01	3.13E-02	1.15E-02
**21**	$CD45RA^+$; $RT1B^+$	0.99 +/-0.25	2.35 +/-0.67	2.62 +/-0.68	7.25E-08	4.42E-06	1.55E-07	4.90E-01
**22**	$CD3^+$; $CD8^+$	3.40 +/-1.16	1.69 +/-0.87	2.59 +/-1.05	1.29E-03	8.44E-04	1.52E-01	9.81E-02
**23**	CD8+;	0.81 +/-0.47	2.15 +/-1.21	1.76 +/-1.50	2.10E-02	1.89E-02	1.23E-01	6.75E-01
**24**	$CD11b/c^+$	ns	ns	ns	ns	ns	ns	ns
**25**	$CD11b/c^+$	1.37 +/-0.39	2.1 +/-0.61	1.67 +/-0.60	5.77E-03	4.30E-03	3.79E-01	1.05E-01
**26**	$CD11b/c^+$	0.62 +/-0.48	1.43 +/-0.65	1.57 +/-0.48	2.68E-04	2.45E-03	4.49E-04	8.18E-01
**27**	$CD45RA^+$; $RT1B^+$	1.00 +/-0.29	3.37 +/-0.68	2.26 +/-0.47	2.86E-12	1.33E-12	2.01E-06	1.79E-05
**28**	$CD45RA^+$; $RT1B^+$	0.88 +/-0.19	2.24 +/-0.72	2.18 +/-0.54	2.34E-07	1.28E-06	2.85E-06	9.60E-01
**29**	$CD161a^+$	0.96 +/-0.21	1.04 +/-0.24	1.25 +/-0.23	1.00E-02	6.92E-01	9.65E-03	6.71E-02
**30**	$CD8^+$; $CD11b/c^+$	2.55 +/-1.37	1.61 +/-1.04	1.27 +/-1.08	3.27E-02	1.40E-01	3.12E-02	7.61E-01
**31**	$CD11b/c^+$	ns	ns	ns	ns	ns	ns	ns

32	CD11b/c^{+}	0.44 +/-0.09	2.89 +/-0.73	1.86 +/-0.50	1.70E-12	8.62E-13	3.12E-07	6.76E-05
33	CD11b/c^{+}	1.11 +/-0.29	3.39 +/-0.94	2.12 +/-0.38	9.08E-10	4.58E-10	8.47E-04	4.29E-05
34	CD45RA^{+}; RT1B^{+}	2.47 +/-0.81	1.78 +/-0.54	1.44 +/-0.28	5.29E-04	1.87E-02	4.19E-04	3.44E-01
35	CD45RA^{+}; RT1B^{+}	1.31 +/-0.14	3.51 +/-0.48	2.71 +/-0.51	6.91E-14	4.30E-14	4.45E-09	1.06E-04
36	CD8^{+}; CD161a^{+}; Tbet^{+}	1.31 +/-0.18	1.94 +/-0.21	2.00 +/-0.45	3.98E-06	5.63E-05	1.14E-05	8.51E-01
37	CD8^{+}; CD161a^{+}	1.62 +/-0.39	1.02 +/-0.55	0.54 +/-0.14	8.06E-07	2.08E-03	4.46E-07	1.70E-02
38	CD11b/c^{+}	4.18 +/-1.29	1.31 +/-0.99	1.81 +/-0.91	3.13E-07	5.88E-07	1.68E-05	4.94E-01
39	CD11b/c^{+}	3.15 +/-1.33	2.53 +/-0.67	1.89 +/-0.60	8.49E-03	2.45E-01	6.00E-03	2.24E-01
40	CD11b/c^{+}	ns	ns	ns	ns	ns	ns	ns
41	CD45RA^{+}; RT1B^{+}	2.27 +/-0.46	2.85 +/-0.49	2.33 +/-0.49	9.78E-03	1.43E-02	9.37E-01	3.25E-02
42	CD45RA^{+}; RT1B^{+}	3.49 +/-0.81	4.04 +/-0.85	3.23 +/-0.41	2.94E-02	1.64E-01	6.57E-01	2.55E-02
43	CD8^{+}; CD161a^{+}; Tbet^{+}	2.19 +/-0.52	1.01 +/-0.33	1.56 +/-0.46	1.09E-06	5.87E-07	3.89E-03	1.21E-02
44	CD8^{+}; CD161a^{+}; Tbet^{+}	1.06 +/-0.43	1.34 +/-0.47	1.81 +/-0.83	1.56E-02	4.94E-01	1.23E-02	1.54E-01
45	CD11b/c^{+}	ns	ns	ns	ns	ns	ns	ns
46	CD11b/c^{+}	5.58 +/-2.53	0.92 +/-0.88	1.93 +/-1.21	2.36E-07	3.28E-07	2.49E-05	3.14E-01
47	CD161a^{+}	1.96 +/-0.31	1.12 +/-0.26	1.18 +/-0.28	1.33E-08	6.67E-08	3.21E-07	8.48E-01
48	CD45RA^{+}; RT1B^{+}	3.70 +/-1.50	1.23 +/-0.99	2.70 +/-1.52	3.91E-04	2.61E-04	1.82E-01	3.23E-02
49	CD45RA^{+}; RT1B^{+}	2.56 +/-0.66	3.03 +/-0.78	3.62 +/-0.83	6.84E-03	2.94E-01	4.86E-03	1.60E-01
