# Supplementary material for: Unbiased Screening Identifies Functional Differences in NK Cells After Early Life Psychosocial Stress
Source: Front Immunol. 2021 Jul 30;12:674532. doi: 10.3389/fimmu.2021.674532 (PMC8363253; doi:10.3389/fimmu.2021.674532)
Supplement: Supplementary file 2 [file Presentation_1.pptx]

## Slide 1
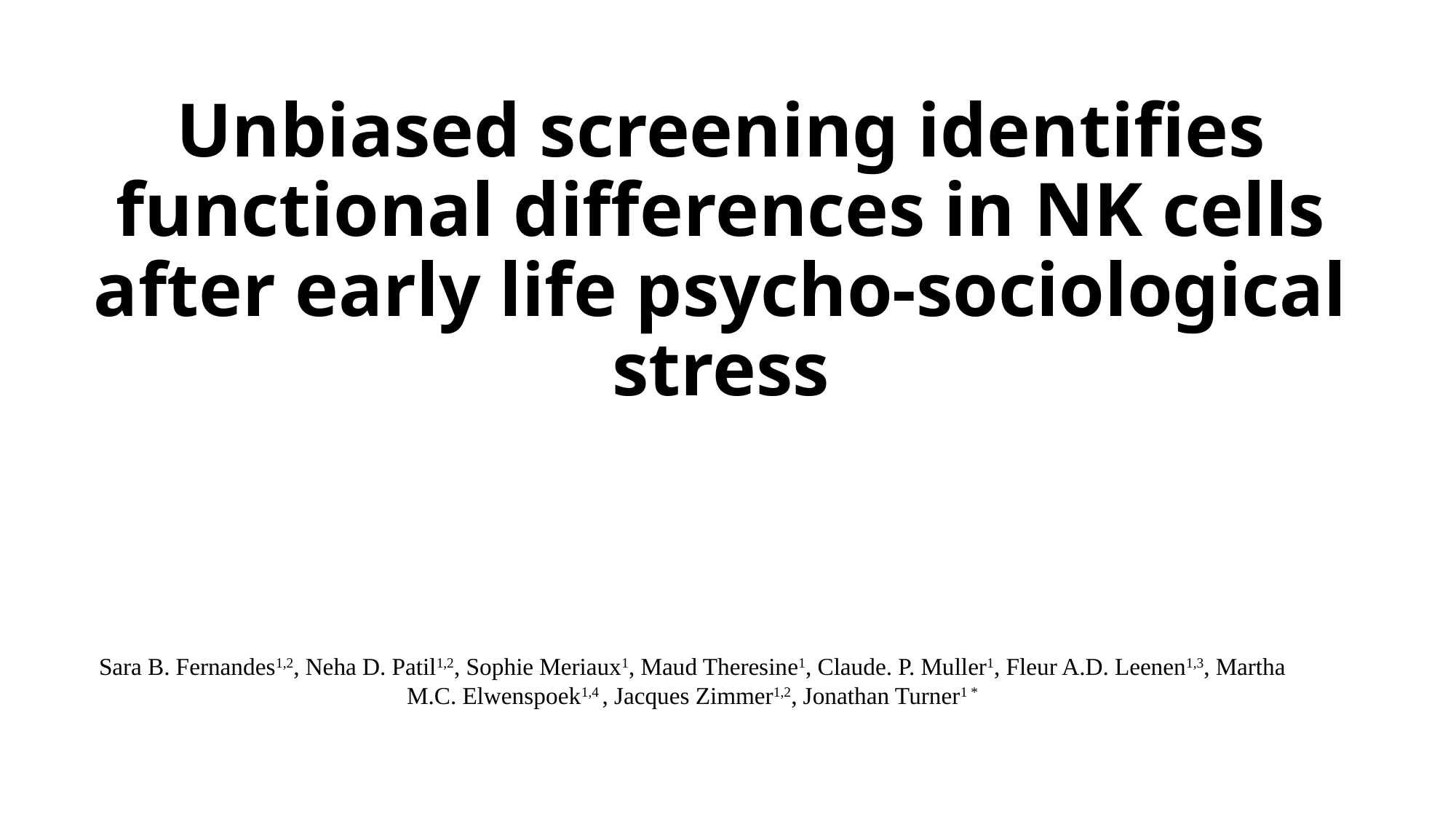

# Unbiased screening identifies functional differences in NK cells after early life psycho-sociological stress
Sara B. Fernandes1,2, Neha D. Patil1,2, Sophie Meriaux1, Maud Theresine1, Claude. P. Muller1, Fleur A.D. Leenen1,3, Martha M.C. Elwenspoek1,4 , Jacques Zimmer1,2, Jonathan Turner1 *

## Slide 2
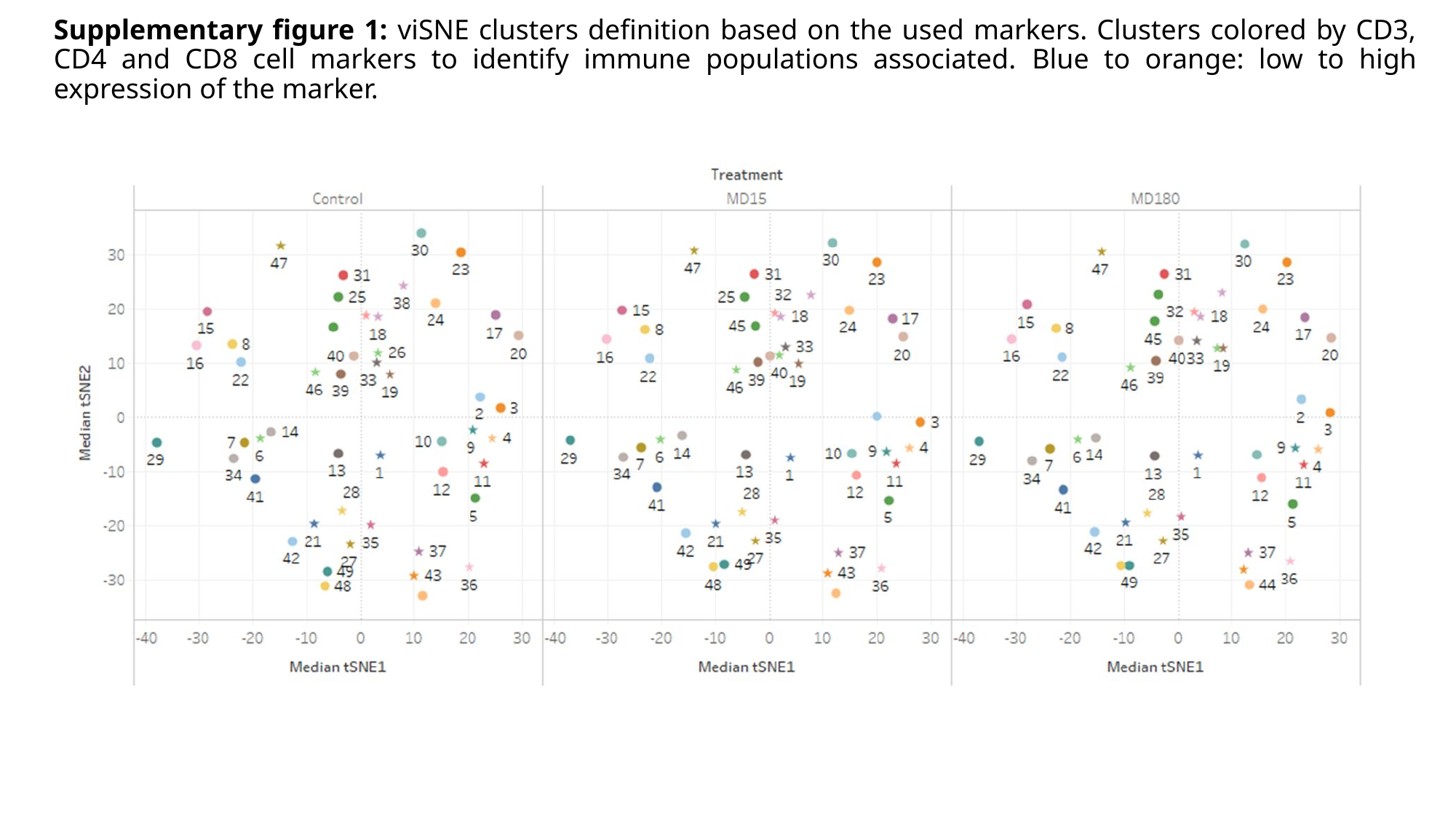

# Supplementary figure 1: viSNE clusters definition based on the used markers. Clusters colored by CD3, CD4 and CD8 cell markers to identify immune populations associated. Blue to orange: low to high expression of the marker.

## Slide 3
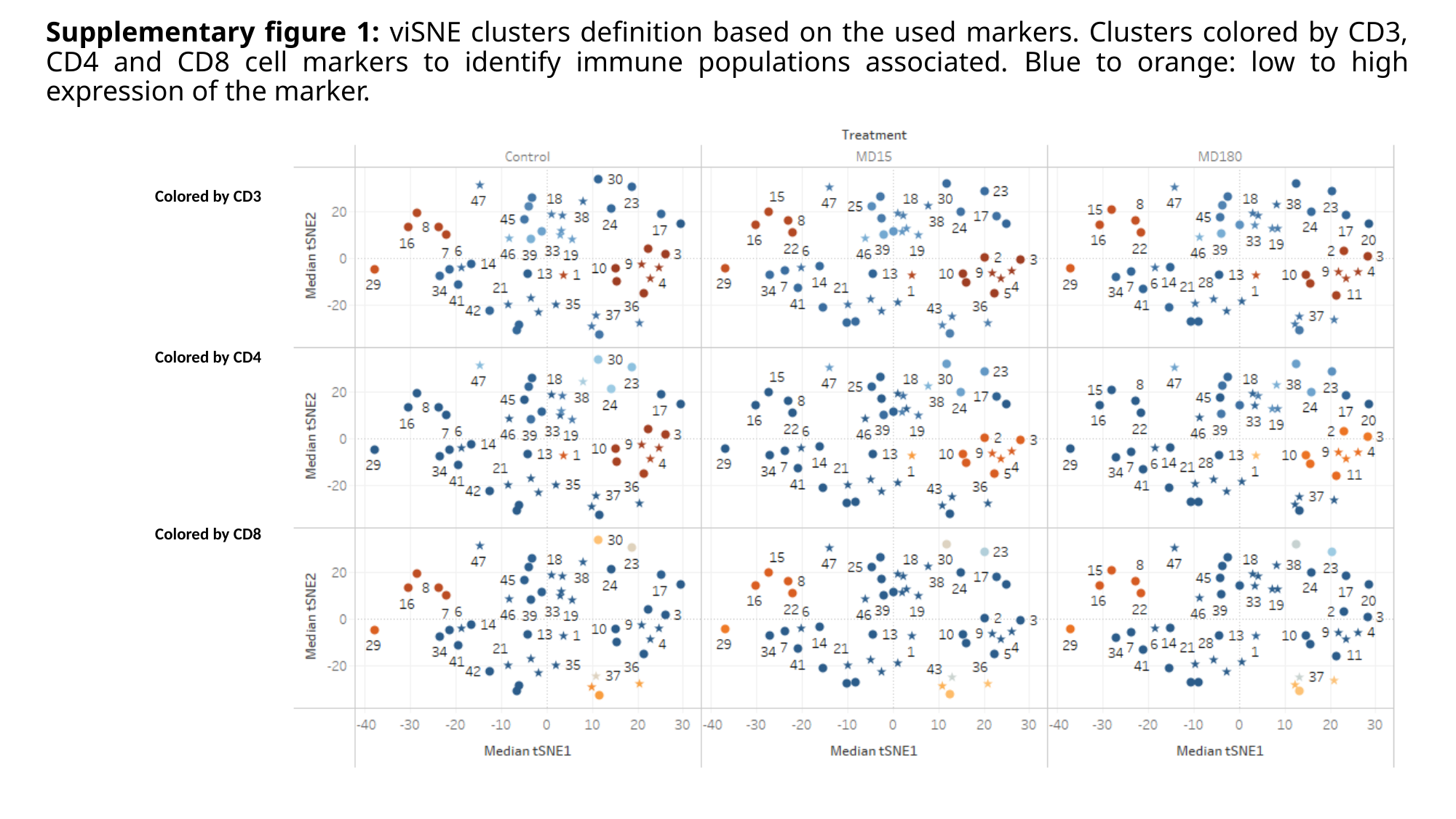

# Supplementary figure 1: viSNE clusters definition based on the used markers. Clusters colored by CD3, CD4 and CD8 cell markers to identify immune populations associated. Blue to orange: low to high expression of the marker.
Colored by CD3
Colored by CD4
Colored by CD8

## Slide 4
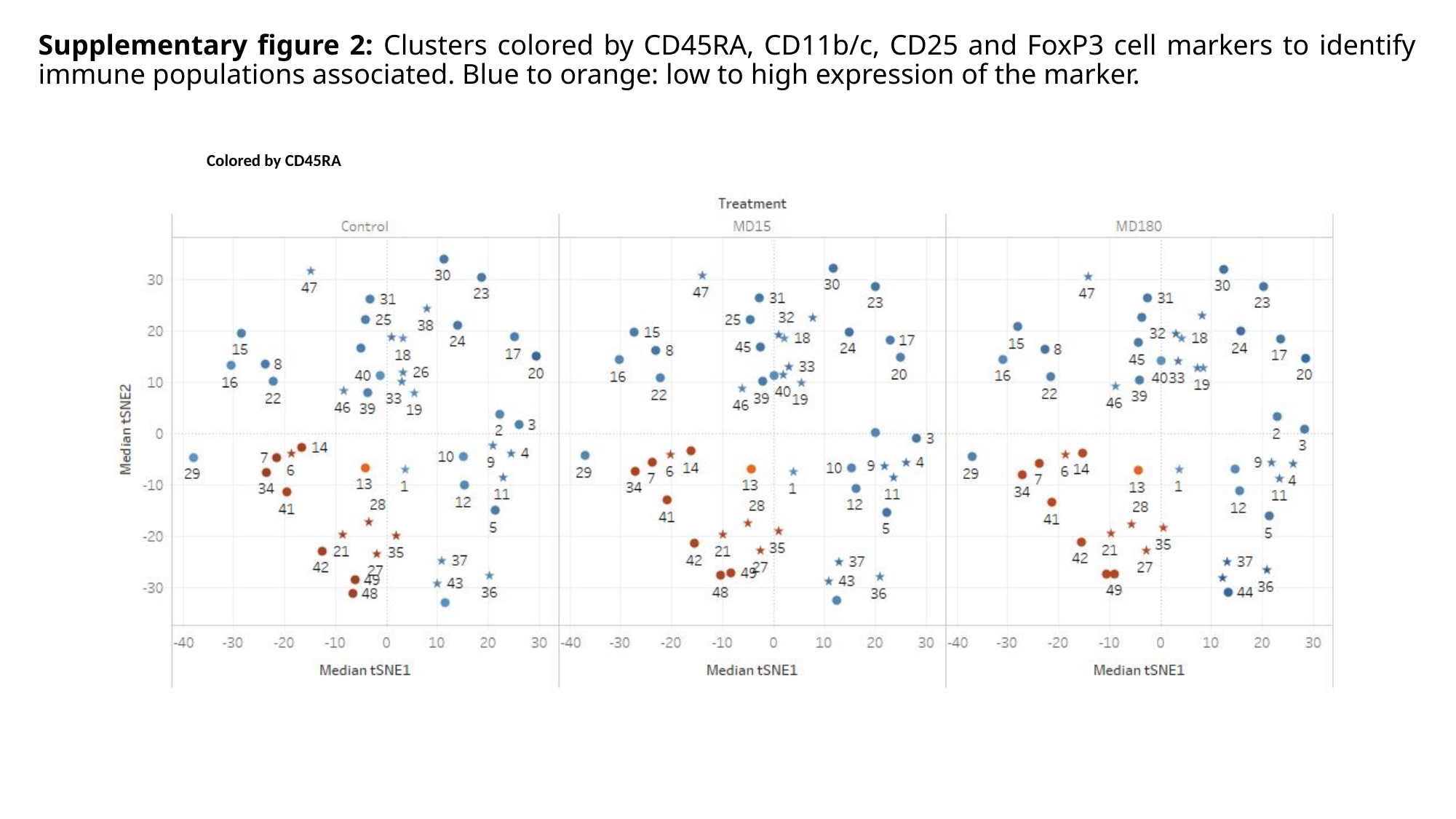

# Supplementary figure 2: Clusters colored by CD45RA, CD11b/c, CD25 and FoxP3 cell markers to identify immune populations associated. Blue to orange: low to high expression of the marker.
Colored by CD45RA

## Slide 5
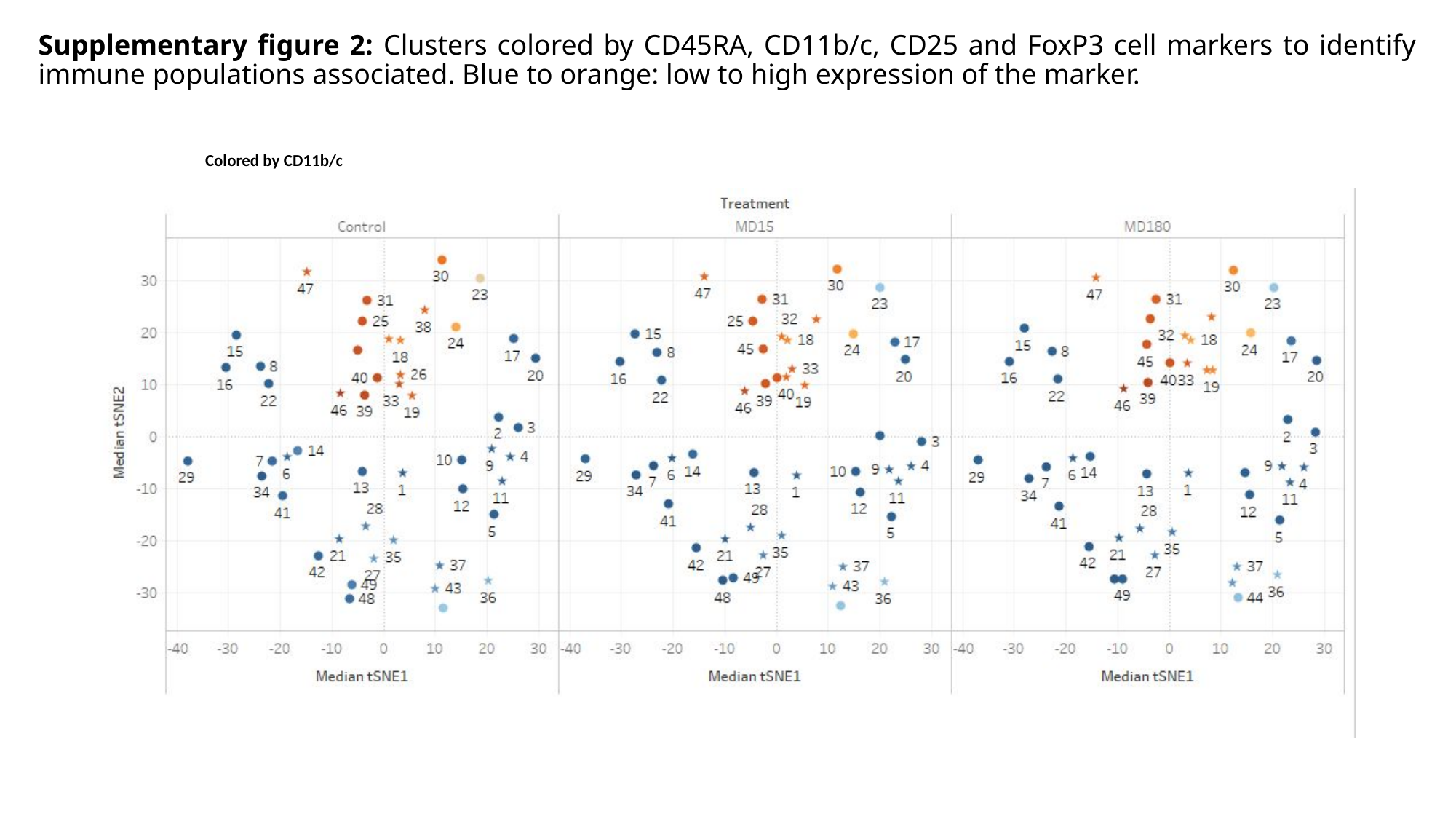

Supplementary figure 2: Clusters colored by CD45RA, CD11b/c, CD25 and FoxP3 cell markers to identify immune populations associated. Blue to orange: low to high expression of the marker.
Colored by CD11b/c

## Slide 6
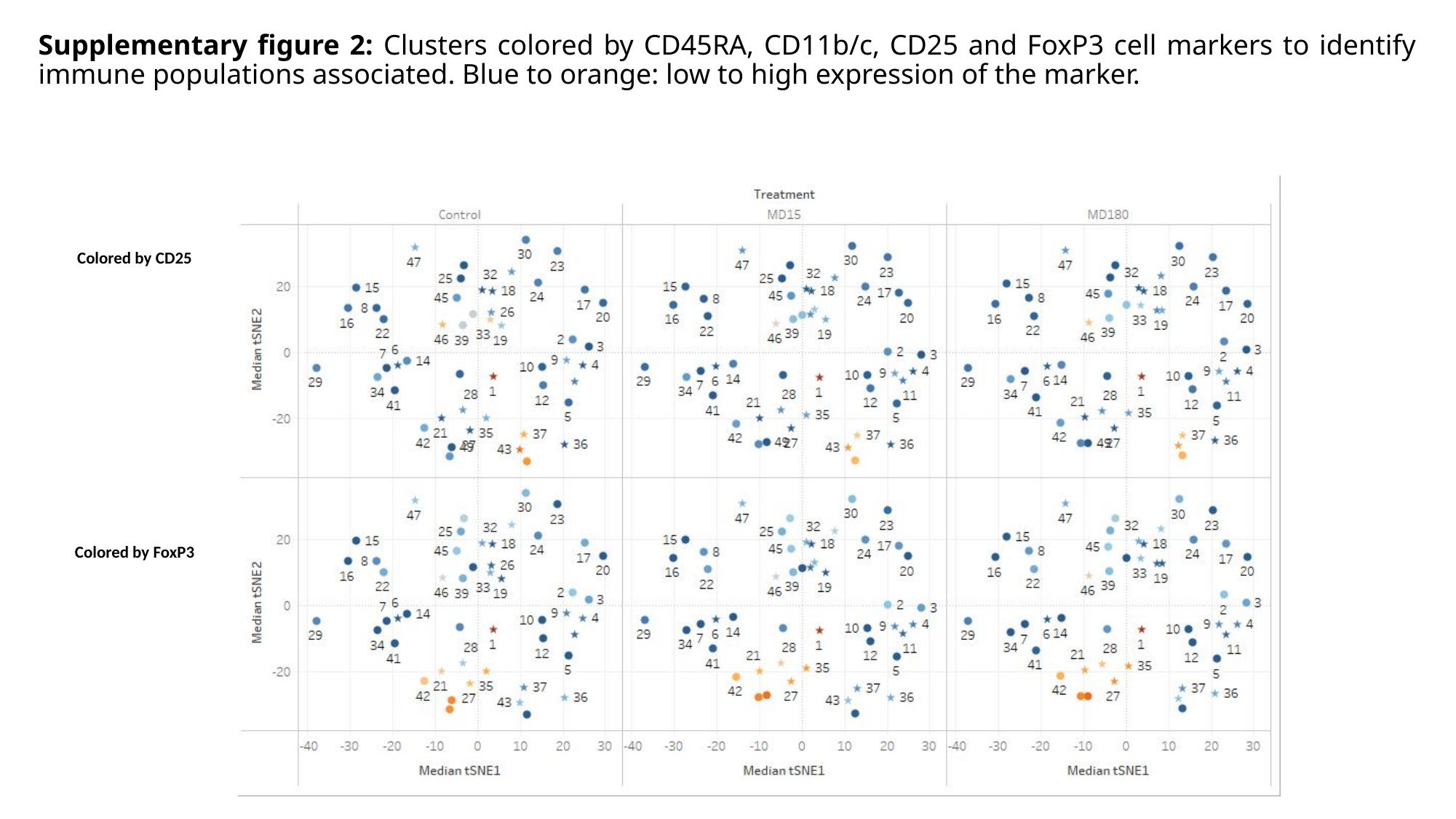

Supplementary figure 2: Clusters colored by CD45RA, CD11b/c, CD25 and FoxP3 cell markers to identify immune populations associated. Blue to orange: low to high expression of the marker.
Colored by CD25
Colored by FoxP3

## Slide 7
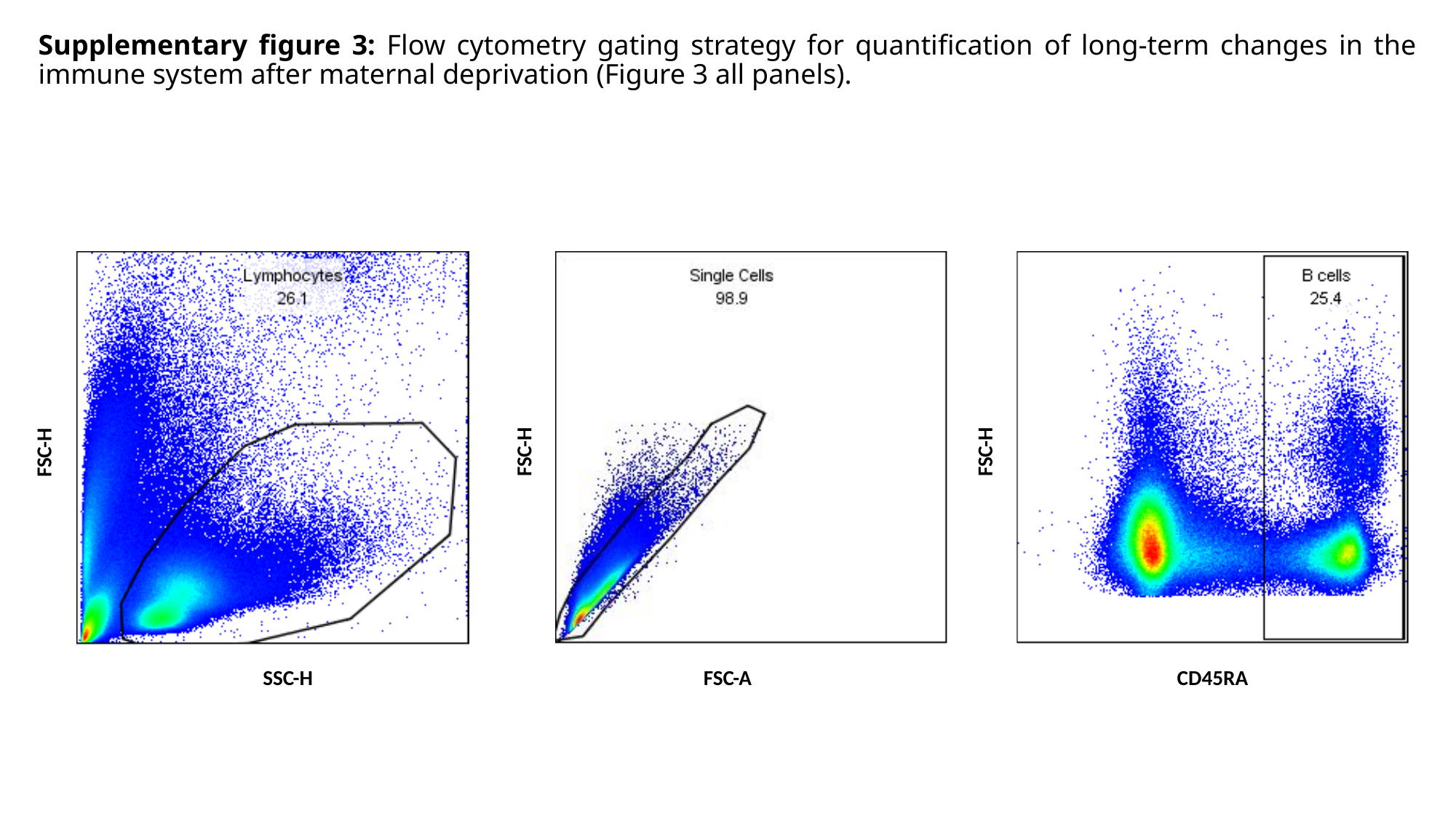

Supplementary figure 3: Flow cytometry gating strategy for quantification of long-term changes in the immune system after maternal deprivation (Figure 3 all panels).
FSC-H
FSC-H
FSC-H
SSC-H
FSC-A
CD45RA

## Slide 8
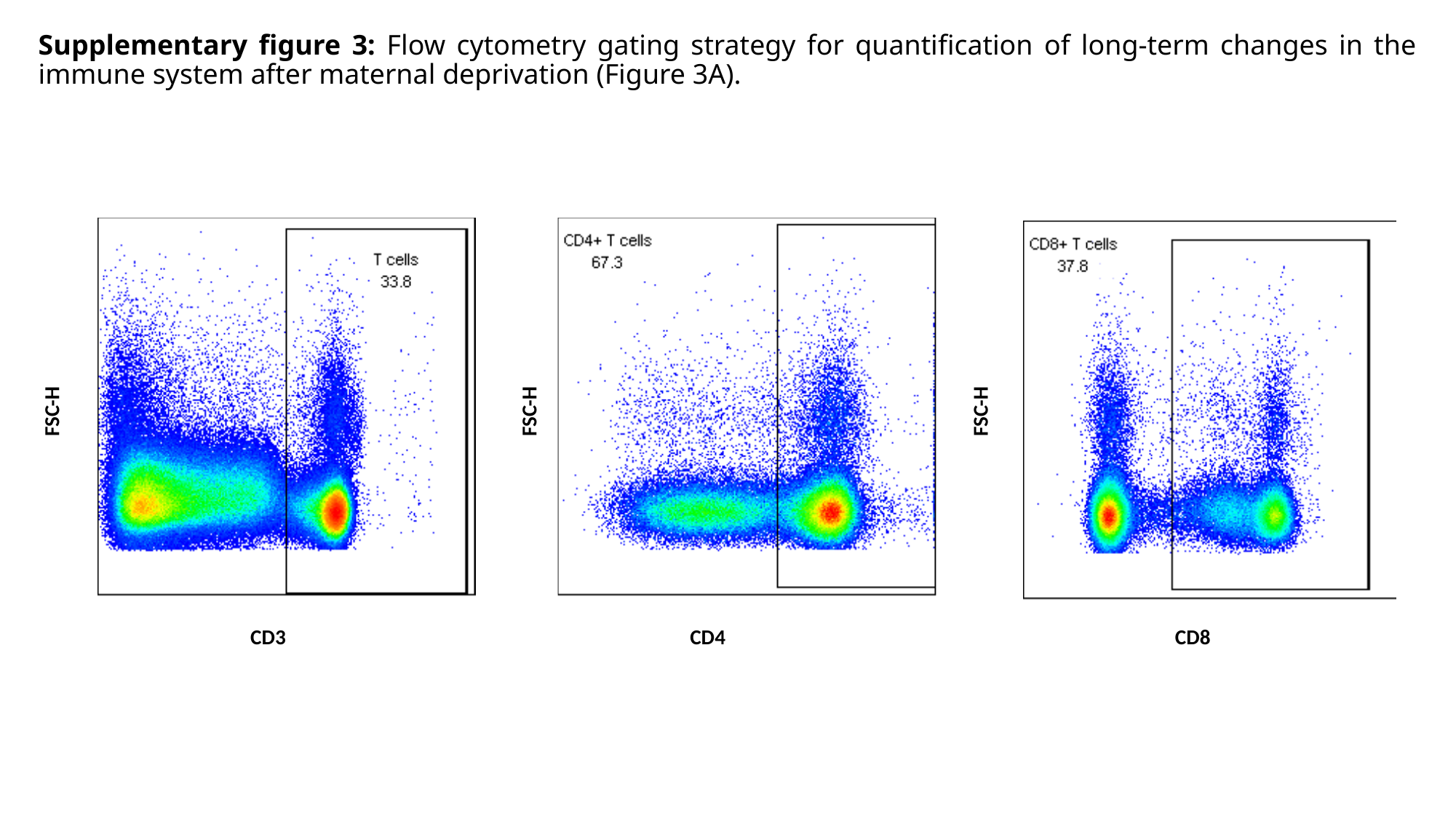

Supplementary figure 3: Flow cytometry gating strategy for quantification of long-term changes in the immune system after maternal deprivation (Figure 3A).
FSC-H
FSC-H
FSC-H
CD3
CD4
CD8

## Slide 9
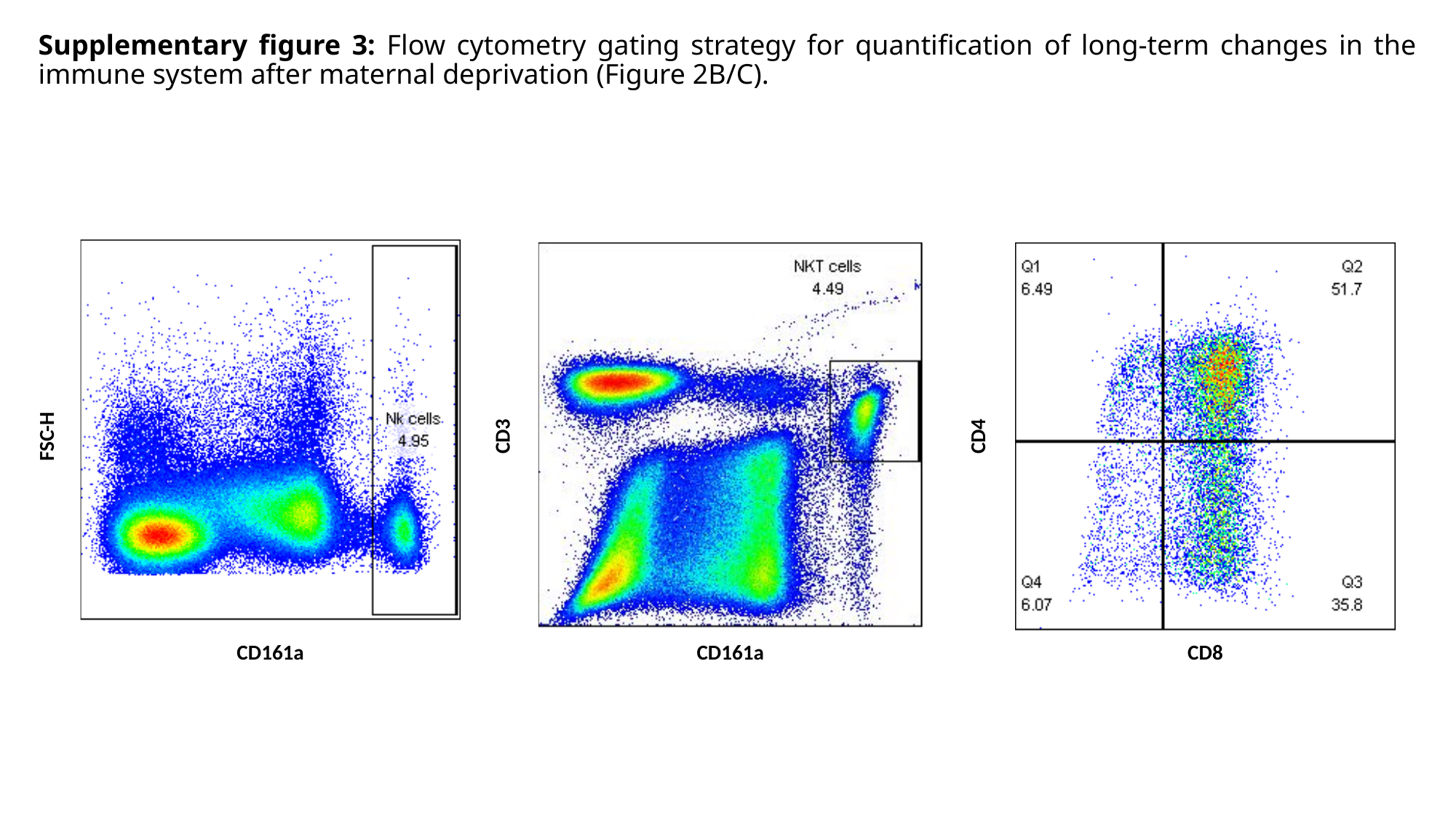

Supplementary figure 3: Flow cytometry gating strategy for quantification of long-term changes in the immune system after maternal deprivation (Figure 2B/C).
FSC-H
CD3
CD4
CD161a
CD161a
CD8

## Slide 10
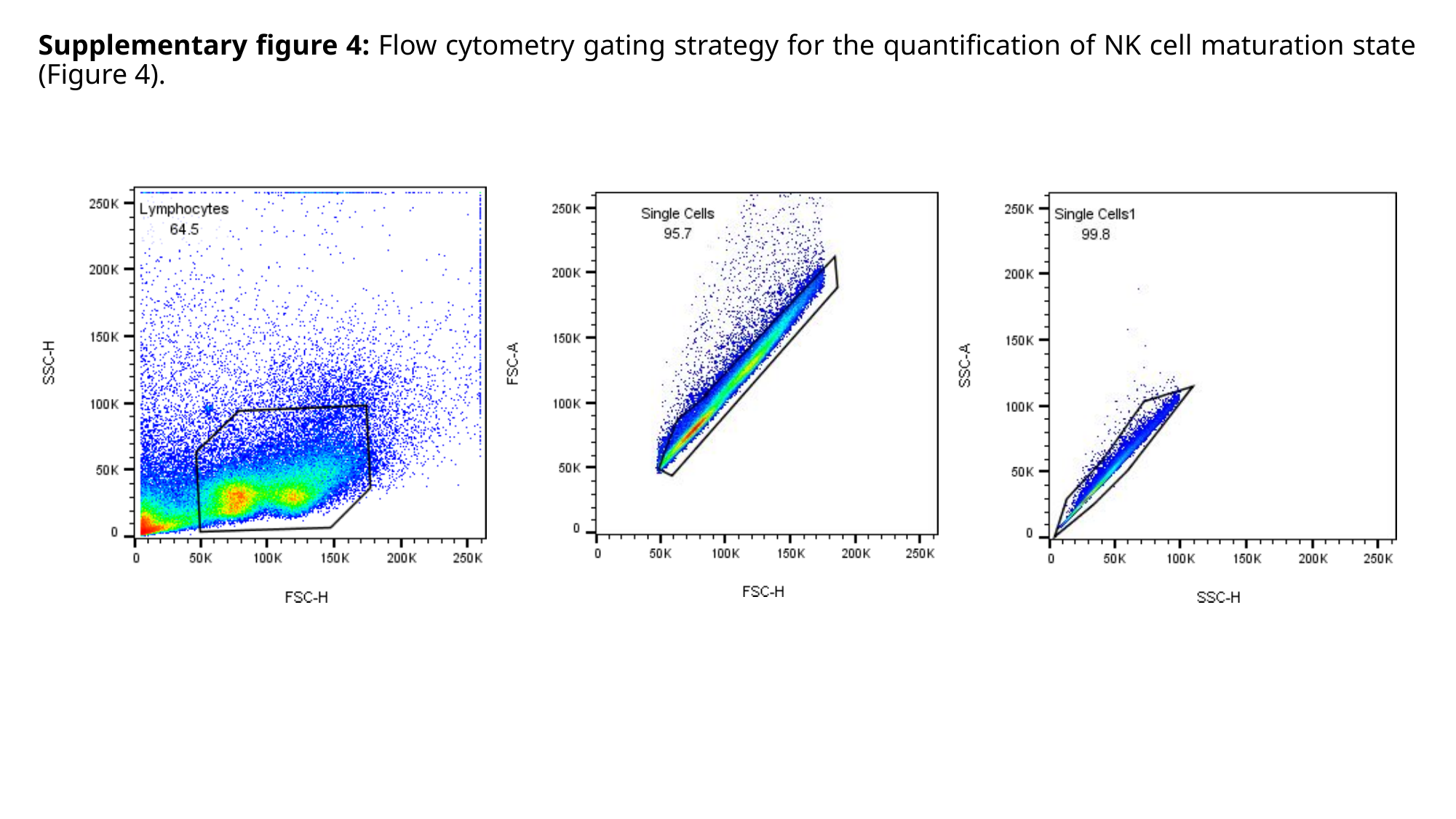

Supplementary figure 4: Flow cytometry gating strategy for the quantification of NK cell maturation state (Figure 4).

## Slide 11
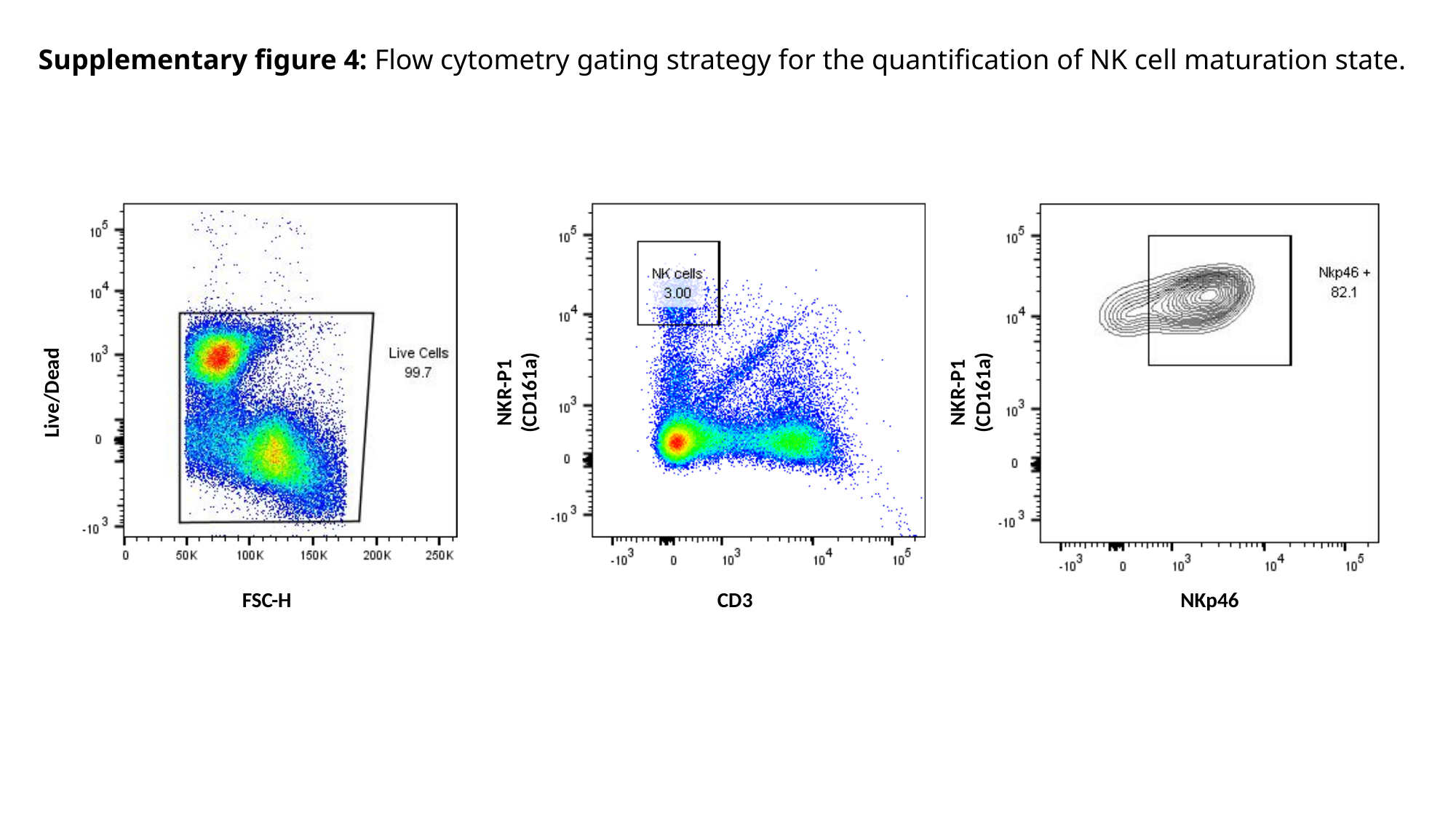

Supplementary figure 4: Flow cytometry gating strategy for the quantification of NK cell maturation state.
Live/Dead
NKR-P1 (CD161a)
NKR-P1 (CD161a)
FSC-H
CD3
NKp46

## Slide 12
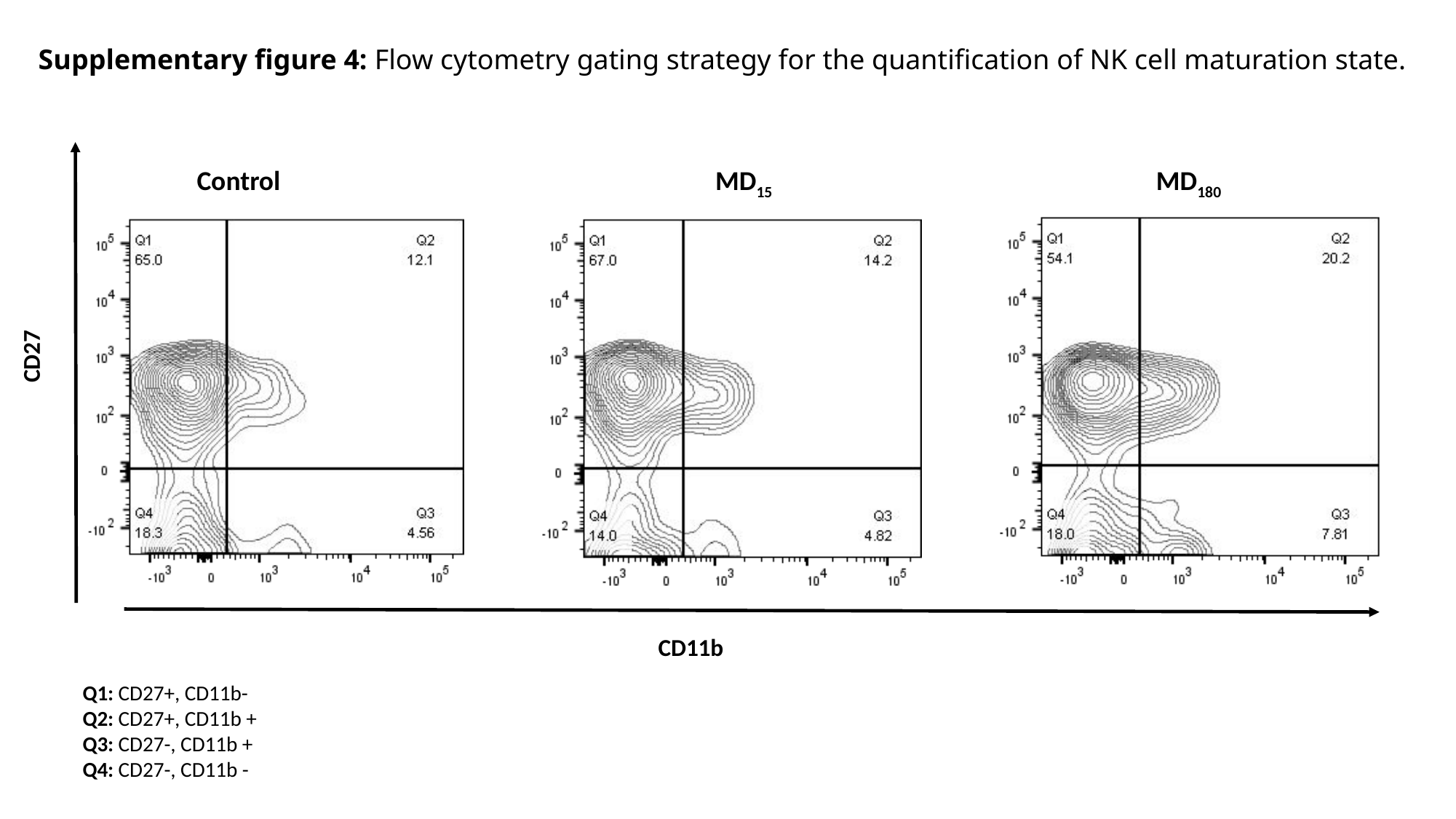

Supplementary figure 4: Flow cytometry gating strategy for the quantification of NK cell maturation state.
Control
MD15
MD180
CD27
CD11b
Q1: CD27+, CD11b-
Q2: CD27+, CD11b +
Q3: CD27-, CD11b +
Q4: CD27-, CD11b -

## Slide 13
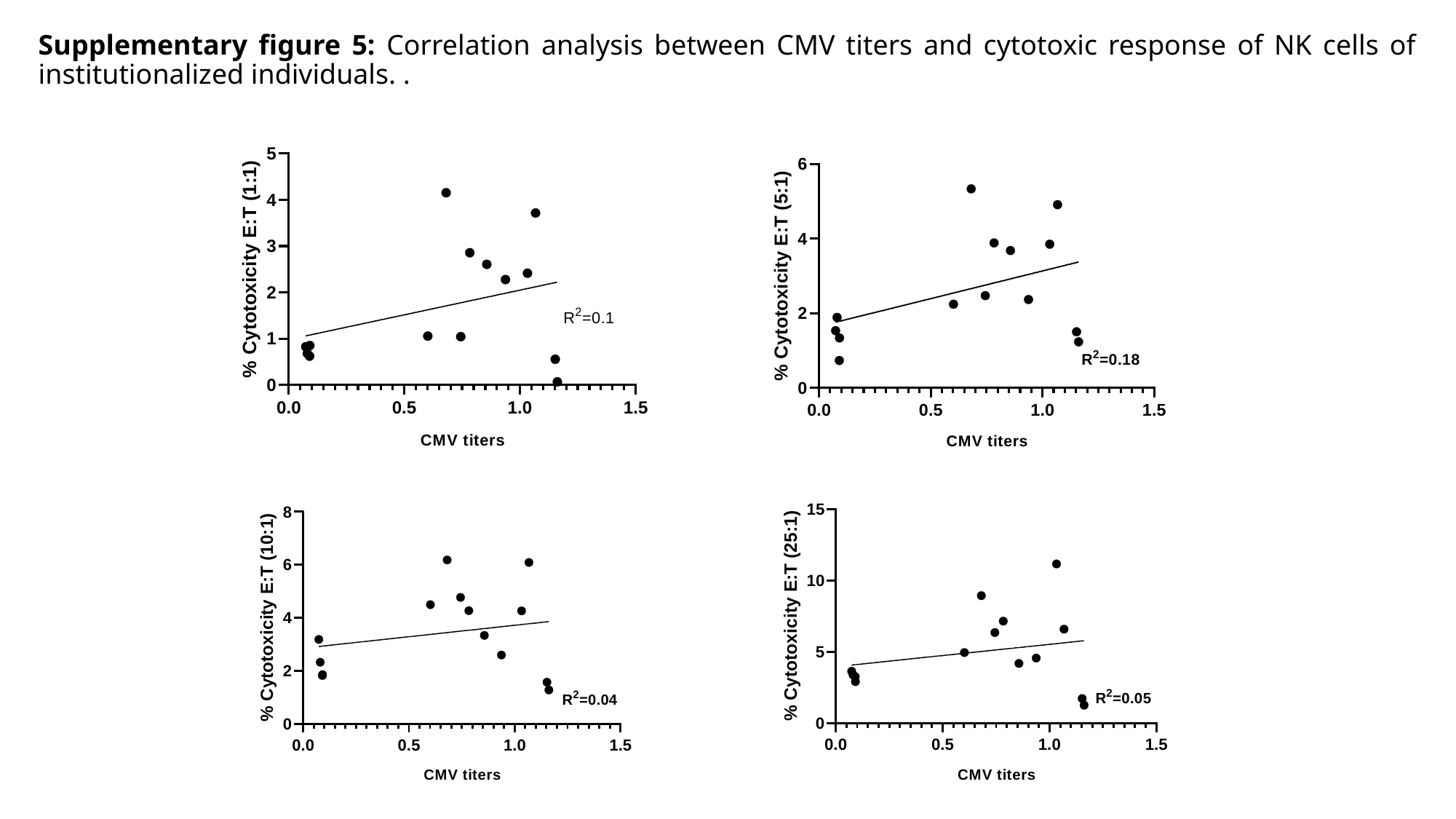

Supplementary figure 5: Correlation analysis between CMV titers and cytotoxic response of NK cells of institutionalized individuals. .

## Slide 14
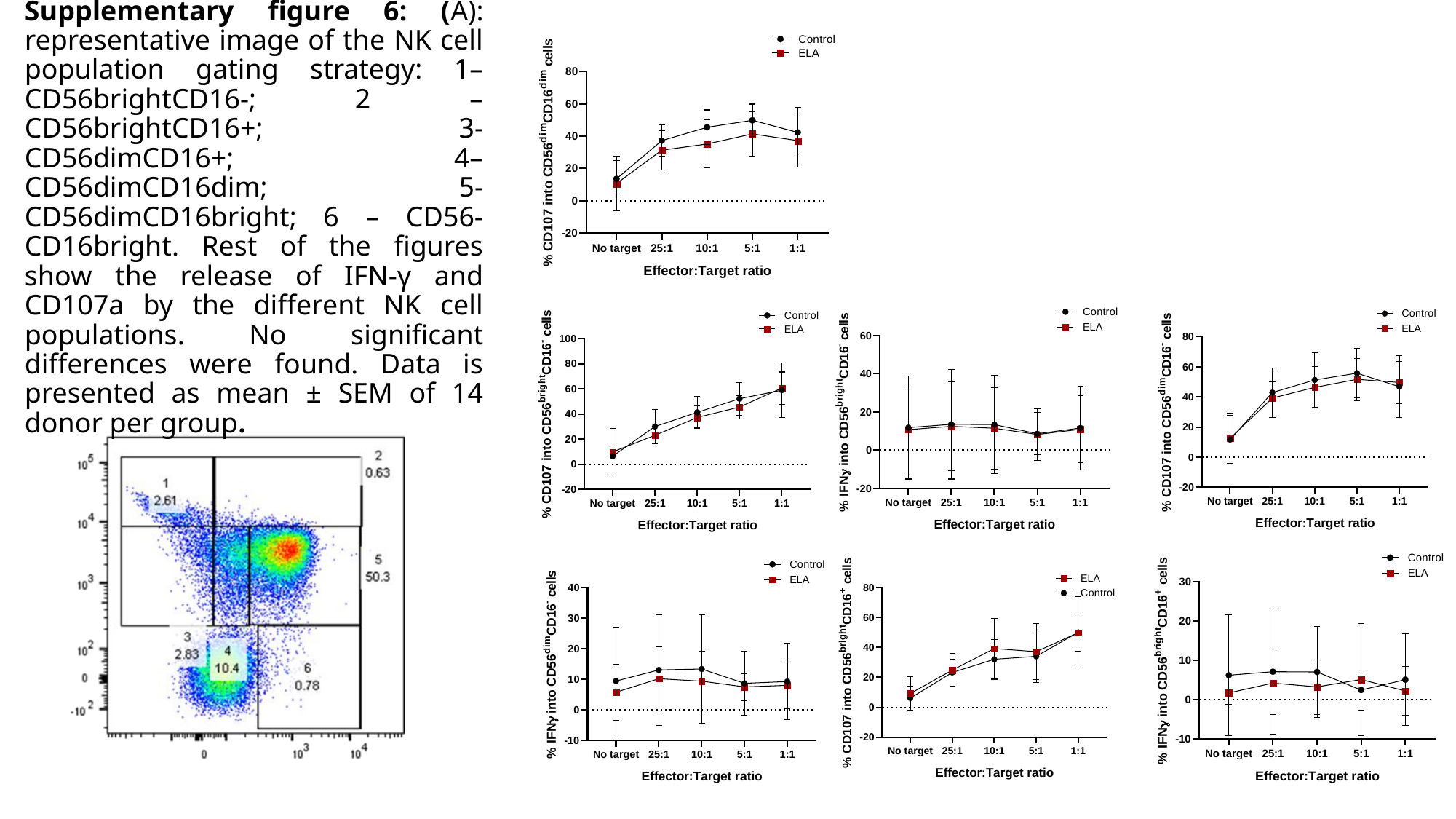

Supplementary figure 6: (A): representative image of the NK cell population gating strategy: 1– CD56brightCD16-; 2 –CD56brightCD16+; 3- CD56dimCD16+; 4– CD56dimCD16dim; 5- CD56dimCD16bright; 6 – CD56-CD16bright. Rest of the figures show the release of IFN-γ and CD107a by the different NK cell populations. No significant differences were found. Data is presented as mean ± SEM of 14 donor per group.
